# Genome-Wide Identification and Expression Pattern Analysis of Nuclear Factor Y B/C Genes in *Pinus koraiensis*, and Functional Identification of *LEAFY COTYLEDON 1*

**DOI:** 10.3390/plants14030438

**Published:** 2025-02-02

**Authors:** Xiuyue Xu, Xin He, Qun Zhang, Ling Yang

**Affiliations:** 1 State Key Laboratory of Tree Genetics and Breeding, Northeast Forestry University, Harbin 150040, China; 15663593162@163.com (X.X.); 18545891296@163.com (X.H.); zqzq19960420@163.com (Q.Z.); 2 College of Forestry, Beijing Forestry University, Beijing 100091, China

**Keywords:** *Pinus koraiensis*, bioinformatics, nuclear factor Y, somatic embryogenesis

## Abstract

The nuclear factor Y (NF-Y) transcription factor is widely involved in various plant biological processes, such as embryogenesis, abscisic acid signaling, and abiotic stress responses. This study presents a comprehensive genome-wide identification and expression profile of transcription factors NF-YB and NF-YC in *Pinus koraiensis*. Eight NF-YB and seven NF-YC transcription factors were identified through bioinformatics analysis, including sequence alignment, phylogenetic tree construction, and conserved motif analysis. We evaluate the expression patterns of NF-YB/C genes in various tissues and somatic embryo maturation processes through the transcriptomics of ABA-treated tissues from multiple nutritional tissues, reproductive tissues, and somatic embryo maturation processes. The *Leafy cotyledon1* (*LEC1*) gene belongs to the LEC1-type gene in the NF-YB family, numbered *PkNF-YB7*. In this study, we characterized the function of PkLEC1 during somatic embryonic development using genetic transformation techniques. The results indicate that PkNF-YB/C transcription factors are involved in the growth and development of nutritional tissues and reproductive organs, with specific high expression in *PkNF-YB7* embryogenic callus, somatic embryos, zygotic embryos, and macropores. Most *PkNF YB/C* genes do not respond to ABA treatment during the maturation culture process. Compared with the absence of ABA, *PkNF-YB8* was up-regulated in ABA treatment for one week (4.1 times) and two weeks (11.6 times). However, *PkNF-YC5* was down-regulated in both one week (0.6 times) and two weeks (0.36 times) of culture, but the down-regulation trend was weakened in tissues treated with ABA (0.72–0.83 times). In addition, the promoter of *PkNF YB/Cs* was rich in elements that respond to various plant hormones, indicating their critical role in hormone pathways. The overexpression of *PkLEC1* stimulated the generation of early somatic embryos from callus tissue with no potential for embryogenesis, enhancing the somatic embryogenesis ability of *P. koraiensis* callus tissue.

## 1. Introduction

Nuclear factor Y (NF-Y) is widespread in plants, animals, and other eukaryotes, and is also termed the CCAAT Binding Factor (CBF) or Heme Activator Protein (HAP) [1]. The individual subunits of NF-Y cannot regulate transcription independently; they must function in heterodimers or heterotrimers [2]. The NF-Y complex consists of the subunits NF-YA (CBF-B/HAP2), NF-YB (CBF-A/HAP3), and NF-YC (CBF-C/HAP5), all of which are necessary for binding to the CCAAT box [2,3]. The structural units of the NF-YA, NF-YB, and NF-YC are dissimilar. The NF-YA subunits are located at the nucleus and predominantly bind to the CCAAT box present in the target gene promoters [4]. NF-YB and NF-YC possess histone-fold motifs that allow them to form a tight dimer. This dimer can then interact with NF-YA. NF-YA [5], NF-YB, and NF-Y-C are unable to form homodimer (indicated through yeast two hybrid assays) but have been known to form many heteromeric complexes (NF-YA-C or NF-YC-B) and differentially affect target genes and downstream mechanisms [6]. The NF-Y complex can thus act either as a transcriptional activator or as a repressor, and its activity can be modulated by interaction with other transcription factors (TFs) or regulatory proteins [7,8].

In plants, NF-Y is involved in various biological processes, such as embryogenesis and endosperm formation, seed germination, root morphogenesis, flowering, chloroplast biogenesis and photosynthesis, starch biosynthesis, and fruit ripening [9,10,11,12,13,14,15,16,17,18,19,20,21,22,23,24,25,26,27,28]. NF-Ys have also been involved in abiotic and biotic stress responses like drought stress, temperature salt stress, and microbial stress [29,30,31,32,33,34,35,36,37,38,39,40,41,42,43,44,45,46]. bZIP67 interacts with NF-YB9, and NF-YC2 directly binds to the ABA response elements (ABRE) in the *SUS2* and *CRC* promoter region to activate gene expression for seed development in *A. thaliana* [47]. *LEC1* (*LEAFY COTYLEDON 1*) was the first identified NF-Y subunit actively participating in embryogenesis and post-embryo development in *A. thaliana* [48].

The non-LEC1 members of the NF-YB family of plant regulators have also been studied. *Arabidopsis* plants overexpressing *AtNF-YB1* showed increased tolerance to drought stress compared with wild type [29]. When the orthologous maize gene *ZmNF-YB2* was overexpressed, drought tolerance was also observed, leading to a significant increase in grain yield under water-limiting conditions [49]. In rice, an RNAi construct silencing *OsHAP3A*, *OsHAP3B,* and *OsHAP3C* resulted in the reduced accumulation of nuclear-encoded photosynthesis transcript [24]. In wheat (*Triticum aestivum*), the overexpression of *TaNF-YB3* led to a significant increase in leaf chlorophyll content and photosynthesis. Multiple NF-YC subunits (C1, C3, C4, and C9) and NF-YB subunits (B2 and B3) can interact with CONSTANS (CO) to regulate flowering time [50]. REPRESSOR OF *ga1*-3 (RGA) and RGA-LIKE2 (RGL2) interact with NF-YA2, NF-YB2/B3, or NF-YC3/C4/C9 to regulate gibberellin (GA) and photoperiod-mediated flowering time, or abscisic acid (ABA) and GA signaling pathways during seed germination, respectively [15,21].

*P. koraiensis* is an important nut economic forest and timber tree species in China, as well as an important greening tree species for landscaping and urban green spaces, with significant economic and ecological value [51]. Due to issues such as the long production cycle and unstable seed yield of *P. koraiensis*, the widespread application of its improved varieties in production is limited, making large-scale propagation challenging [52]. Somatic embryogenesis of plants is currently one of the widely used biotechnologies [53,54,55]. Somatic embryogenesis has the advantages of genetic stability, fast propagation rate, and high survival rate, and is one of the effective means of plant regeneration using biotechnology methods [56,57,58]. At the same time, the pathway of somatic embryogenesis is also a model system for studying the physiological processes, metabolic pathways, gene functions, and molecular regulatory mechanisms of embryonic development [59].

LEC1 belongs to the NUCLEAR FACTOR-Y B family, a key participant in the regulation of somatic and zygotic embryonic development in plants. LEC1 is crucial for the development of zygotic and somatic embryos [9]. LEC1 can activate the LEC1-ABI3-FUS3-LEC2 network in the embryo, thereby regulating embryonic maturation [47]. In Scots pine, the LEC1 gene is relatively highly expressed in the proliferation and late-maturation stages during somatic embryogenesis, while the expression pattern in zygotic embryos is different, with higher expression levels in the proliferation stage followed by a gradual decrease in expression [60].

In this study, a total of eight *PkNF-YB* and seven *PkNF-YC* transcription factors were identified in *P. koraiensis* through the whole genome. We analyzed their protein sequences, chromosome locations, evolutionary relationships, basic physiochemical properties, and gene structures. The expression patterns of these genes in different plant tissues upon nutritional growth, reproductive growth stages, and ABA treatment were also investigated. This study provides a reference for future research on PkNF-YB/C transcription factors and plant stress response. By integrating the somatic embryogenesis and genetic transformation systems of *P. koraiensis*, we investigated the function of the *PkLEC1*. This research complements the molecular level studies on somatic embryogenesis in *P. koraiensis* and offers theoretical and technical support for molecular design breeding aimed at enhancing the yield and quality of somatic embryos in this species.

## 2. Results

### 2.1. Identification and Sequence Analysis of PkNF-YB/C Genes in P. koraiensis

Based on the CBFD_NFYB_HMF (PF00808) conserved domain specific to the NF-YB and NF-YC family members in plant, eight members of the NF-YB and seven members of the NF-YC family members were identified from *P. koraiensis*, and they were named PkNF-YB 1–8 and PkNF-YC 1–7, respectively, according to their locations on chromosomes I to XI. The details of these 15 *PkNF-YB/C* genes, including locus name, chromosome location, number of amino acids, molecular weight, isoelectric point, aliphatic index, and GRAVY score are listed in Table 1. The putative PkNF-YB protein sequences contained 78–225 amino acids and had isoelectric points ranging between 4.96 and 7.76, while putative PkNF-YC protein sequences contained 202–311 amino acids and had isoelectric points ranging between 5.96 and 8.90. All were determined to be hydrophilic proteins.

### 2.2. Phylogenetic Analysis of Multiple Species, Protein Sequence Alignment, and Conservative Motif Analysis of PkNF-YB/Cs

**Figure 1:**
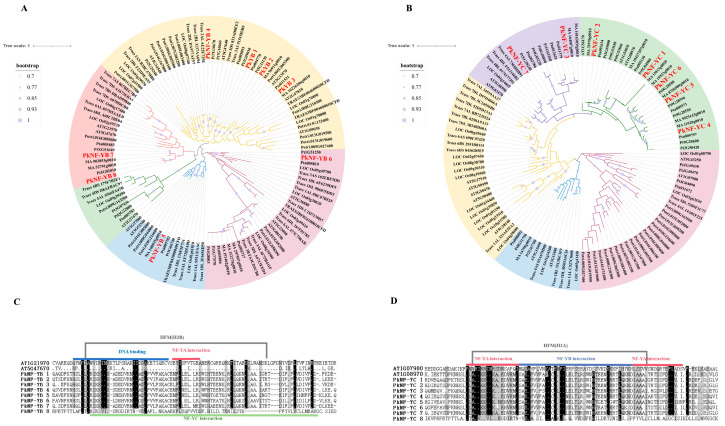
Phylogenetic analysis and multiple alignment of amino acid sequences. Multispecies phylogenetic analysis of NF-YB (**A**) and NF-YC (**B**) proteins, including *Arabidopsis thalian*, *Pinus koraiensis*, *Pinus taeda*, *Pinus tabuliformis*, *Populus trichocarpa*, *Picea abies*, *Oryza sativa*, and *Triticum aestivum*. Amino acid alignment of conserved domains of NF-YB (**C**) and NF-YC (**D**) proteins from different organisms. The sequence under the red line represents the region that interacts with NF-YA, while the sequence under the blue line represents the region that interacts with NF-YB; the sequence above the green line represents the region that interacts with NF-YC, while the sequence below the cyan line represents the region that binds to DNA. Gray lines include segments with conserved HFM regions.

The NF-YB/C amino acid sequences of multiple species were aligned and a phylogenetic tree was constructed (Figure 1A,B). Phylogenetic trees were constructed, and intron and exon structures were compared to further understand the relationship between the members of the *PkNF YB/C* gene families. The *NF-YB* family members were divided into five subfamilies, with the pink subfamily branch being the LEC-type NF-YBs, and the other branches (green, blue, yellow, and pinky purple) were non-LEC-type NF-YBs. The NF-YC family members were divided into five branches, with seven members of *P. koraiensis* concentrated in the green and purple branches. Multiple alignment of protein sequences shows that NF-YB members contain a conserved NF-YA interacting region, a NF-YC interacting region, DNA-binding region, and HFM (H2B) conserved domains (Figure 1C). NF-YC members contain conserved NF-YA interacting regions, NF-YB interacting regions, DNA-binding regions, and HFM (H2A) conserved domains (Figure 1D).

**Figure 2:**
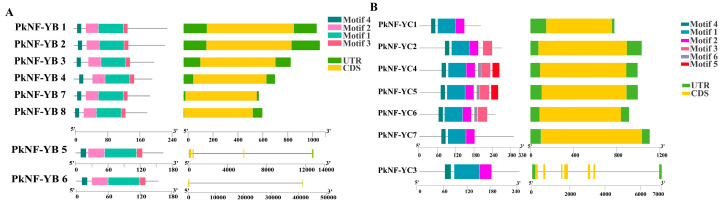
Structural analysis of proteins and conserved structures encoding corresponding proteins. Predicted conserved motifs and structures of PkNF-YBs (**A**) and PkNF-YCs (**B**). Different colors represent different conserved motifs. Yellow represents the protein-coding sequence (exon); green represents upstream/downstream sequences; the black line represents introns.

The conserved motifs of the PkNF-YB/C family were analyzed using the online tool MEME and a schematic map was constructed to represent the structure of the proteins (Figure 2). The PkNF-YB family members have predicted four conserved motifs, each containing these four motifs with the same arrangement. However, NF-*YB1*, *2*, *3*, *4*, *7*, and *8* members do not contain introns, while *NF-YB5* and *6* have ultra-long introns (Figure 2A). The PkNF-YC family members have predicted six conserved motifs, each containing motif 1, 2, 3, and 4. And PkNF-YC2, 4, 5, and 6 members contain motif 3; PkNF-YC4 and 5 members contain motif 5; PkNF-YC4, 5, and 6 members contain motif 6. Similarly, *NF-YC1*, *2*, *4*, *5*, *6*, and *7* members do not contain introns, while *NF-YB3* has ultra-long introns (Figure 2B). The sequences of motifs are shown in Table S3.

### 2.3. Chromosomal Location, Collinearity Analysis, and Ka/Ks Calculation of PkNF-YB/C Genes

**Figure 3:**
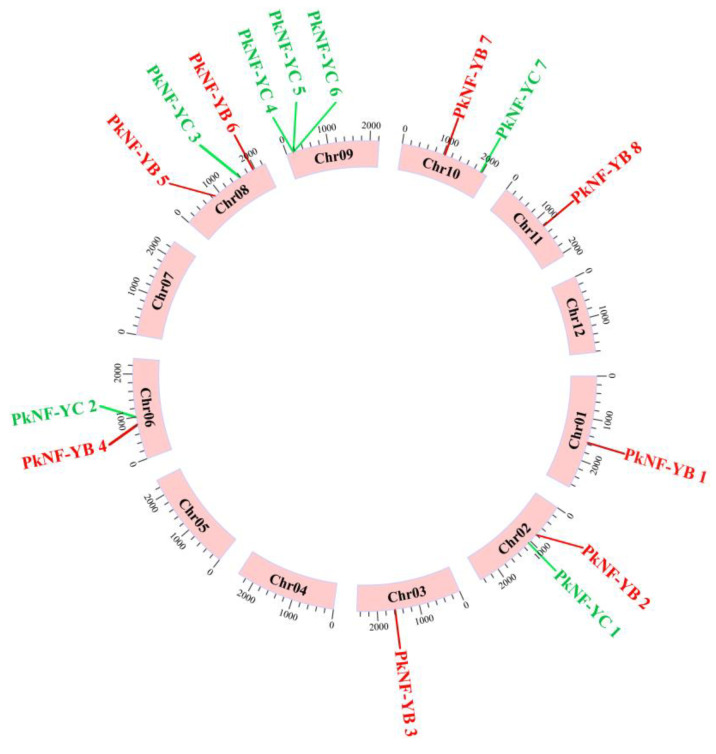
Chromosomal location and intra species collinearity analysis of PKNF-YB/C. Thick red lines indicate duplicated gene pairs. The red font represents members of the NF-YB family, while the green font represents members of the NF-YC family. Chromosome numbers were displayed in the outer ring of each chromosome. There is no collinearity between *NF-YB* and *NF-YC* family members within this species. Scale bars marked on each chromosome indicate chromosome length (Mb).

To determine the distribution of the *PkNF-YB/C* genes, we mapped their positions on the chromosomes. The eight *PkNF-YB* genes were distributed on seven chromosomes: Chr 01, Chr 02, Chr 03, Chr 06, Chr 08, Chr 10, and Chr 11. The seven *PkNF-YC* genes were distributed on five chromosomes: Chr 02, Chr 06, Chr 08, Chr 09, and Chr 10 (Figure 3).

**Figure 4:**
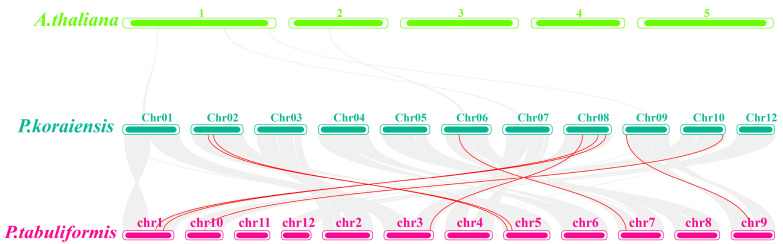
The genomes of *P. koraiensis*, *A. thaliana*, and *P. tabulaeformis* were subjected to collinearity analysis. The highlighted red line represents the *NF-YB/C* gene pairs with collinearity.

We first conducted collinearity analysis within *P. koraiensis* and found that there was no collinearity between the NF-YB and NF-YC family members (Figure 3). Then, we downloaded the genome data of *Arabidopsis thaliana* and *Pinus tabulaeformis* and conducted collinearity analysis between the genomes of *P. koraiensis*, *A. thaliana*, and *P. tabulaeformis*. The NF-YB/C family members of *P. koraiensis* do not have collinear relationships in *Arabidopsis*, but *PkNF-YB2,4,5,6* and *PkNF-YC1,3,7* have collinear members in *P. koraiensis* (Figure 4). The gene pairs with collinearity between different species are listed in Table S4.

To better understand the evolutionary constraints on the *PkNF-YB/C* gene families, the *Ka*/*Ks* ratios of the *PkNF-YB/C* gene pairs were calculated (Table S5). The selection stress analysis showed that the duplicated gene pairs were mainly under purifying selection (*Ka*/*Ks* < 1.0).

### 2.4. Analysis of Cis-Elements of PkNF-YB/C Promoters

**Figure 5:**
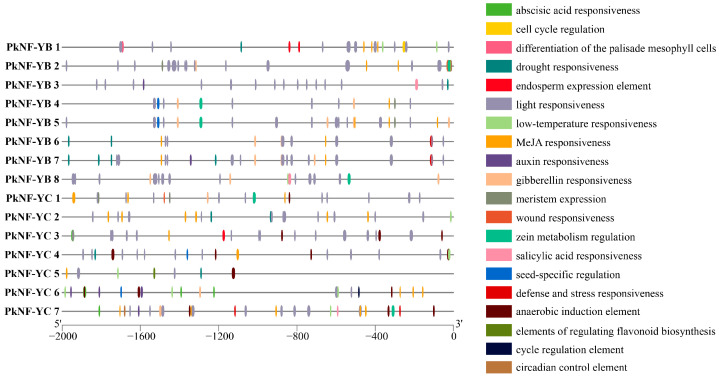
Analysis of *cis*-elements of *PKNF-YB/C* promoters.

*PkNF-YB/C* promoters contain a large number of hormone response elements, such as abscisic acid responsive, auxin responsive, gibberellin responsive, MeJA responsive, and salicylic acid responsive. There are also many abiotic stress response elements, such as low-temperature responsive, drought inducibility, defense and stress responsive, and anaerobic induction. In addition, the largest number was the light-responsive element. In addition, there are also some specific elements related to tissue development and metabolism, such as the meristem expression element, endosperm expression element, seed-specific regulation element, wound-responsive element, cycle regulation element, and zein metabolism regulation element (Figure 5). Thus, we speculate that *PkNF-YB/C* genes may also play an important role in plant growth and development, hormone response, and stress resistance, which adds a new direction to the future research of PkNF-YB/C. The information of the *cis*-acting elements are listed in Table S6.

### 2.5. GO Enrichment and KEGG Enrichment

**Figure 6:**
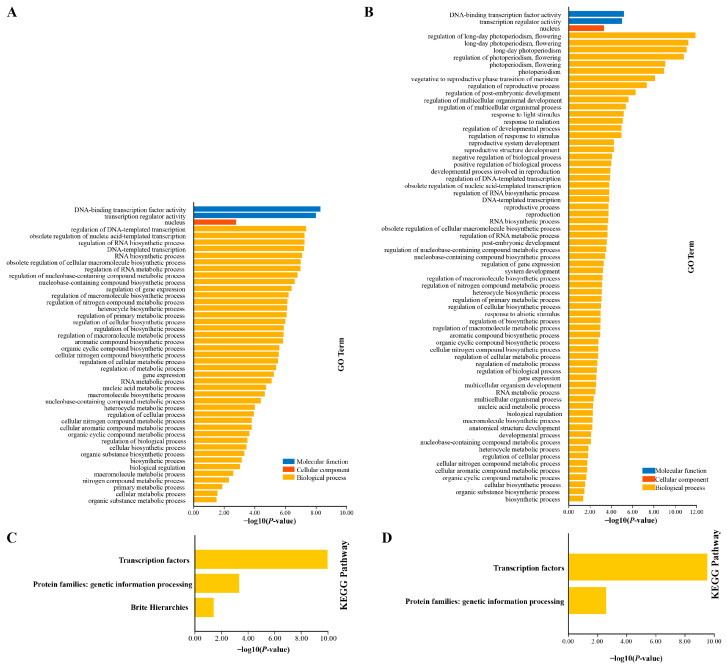
GO enrichment analysis of NF-YB (**A**) and NF-YC (**B**) family members. KEGG enrichment analysis of NF-YB (**C**) and NF-YC (**D**) family members.

In order to further evaluate the biological function of the PkNF-YB/C genes, we conducted GO enrichment and KEGG enrichment analysis on them (Figure 6). In GO enrichment, eight members of PkNF-YB were annotated with two molecular functional entries, one cellular component entry, and forty-two biological process entries (Figure 6A). The seven members of NF-YC were annotated with two molecular functional entries, one cellular component entry, and sixty-six biological process entries (Figure 6B). In KEGG enrichment, eight members of NF-YB were annotated into transcription factors, protein families: genetic information processes, and Brite Hierarchies metabolic pathways (Figure 6C). The seven members of NF-YC were annotated into the transcription factor and protein families: genetic information processes (Figure 6D). This indicates that NF-YB and NF-YC members were widely involved in the metabolic processes of plant growth and development.

### 2.6. Tissue Expression Specificity and Abiotic Stress Expression Pattern

**Figure 7:**
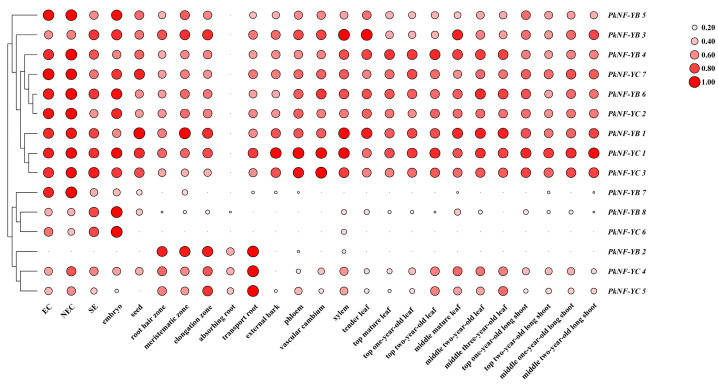
Tissue-specific expression patterns of *PkNF-YB/C* genes in callus, embryo, root, stem, and leaf. EC: embryogenic callus; NEC: non-embryogenic callus; SE: somatic embryogenesis; embryo: zygotic embryo; seed: mature seed. The depth of color and the size of the circle area represent the level of expression. The transcriptome expression data underwent log_2_^(FPKM)^ transformation and were normalized using a 0–1 approach. The circle on the right serves as the scale bar. The larger the area, the redder the color, indicating a higher relative expression level.

As shown in Figure 7, the expression patterns of *PkNF-YB* and *PkNF-YC* members in embryonic callus, non-embryonic callus, somatic embryo, zygotic embryo, seed, external bark, phloem, cambium and xylem, roots, leaves, and branches of different parts were displayed. *PkNF-YB1*, *3*, *4*, *5*, and *6* were expressed in all tissues, while *PkNF-YB2* was up-regulated in the root and almost no expression in other parts. *PkNF-YB7* and *8* were mainly up-regulated during seed development and somatic embryonic development. *PkNF-YC1*, *2*, *3*, and *7* were expressed in all tissues, while *PkNF-YC6* was down-regulated in all tissues. *PkNF-YC4* and *5* were mainly up-regulated during seed and somatic embryonic development, as well as in roots and leaves.

**Figure 8:**
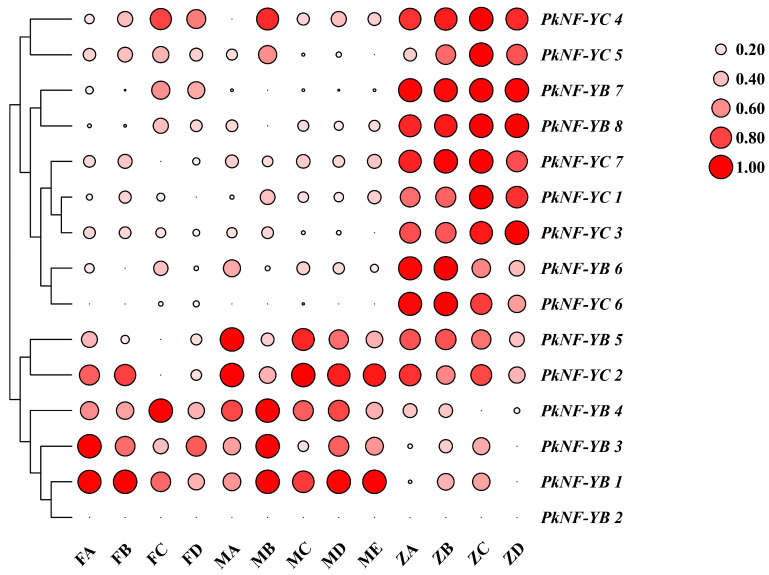
Expression patterns of the *PkNF YB/C* genes at different stages of reproductive organ development. FA–FD indicate different stages of microsporophyll: tender branches without differentiated microsporophyll, immature microsporophyll, mature microsporophyll, and dried and withered microspore leaves after pollen dispersion. MA–ME indicate different stages of megasporophylls: tender branches without differentiated megasporophyll, female flower bud (megasporophyll), closed megasporophylls before pollination, macrosporophyll unfolding during pollination, and closed megasporophylls after pollination. ZA–ZD: ovules during the female gametophyte stage, ovules during pollen tube extension, fertilized ovules, and an ovule that begins cell division and development. The color and the size of the circle area represent the level of expression. The transcriptome expression data underwent log_2_^(FPKM)^ transformation and were normalized using a 0–1 approach. The circle on the right serves as the scale bar. The larger the area, the redder the color, indicating a higher relative expression level.

As shown in Figure 8, the expression patterns of *PkNF-YB* and *PkNF-YC* members in microsporophyll, megasporophyll, and ovules at different developmental stages are displayed. *PkNF-YB1*, *3*, *4*, *5*, and *6* were up-regulated in microsporophyll and megasporophylls, while *PkNF-YB7* and *8* were mainly up-regulated in the middle and late stages of microsporophyll development and ovule development. *PkNF-YB2* and *6* were down-regulated at all stages. *PkNF-YC1* and *7* were up-regulated in all tissues, *PkNF-YC3* and *4* were mainly up-regulated during the development of microspores and ovules, while *PkNF-YC2* was mainly up-regulated during the development of microsporophyll and megasporophylls. *PkNF-YC5* was mainly up-regulated in the early stage of megasporophyll development and the middle to late stage of ovule development, while *PkNF-YC6* was down-regulated only in the early stage of ovule development.

**Figure 9:**
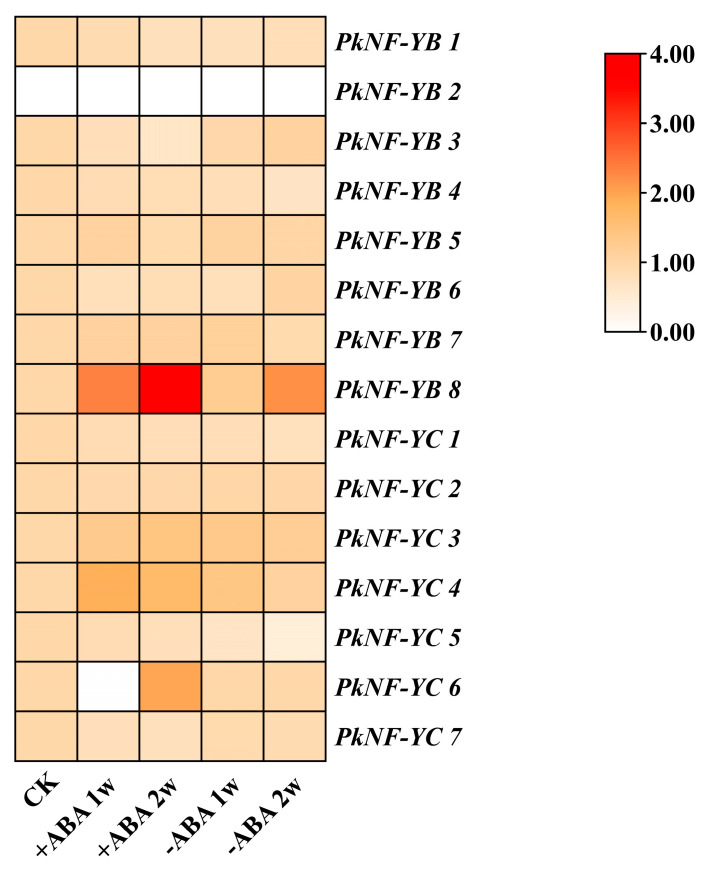
Expression patterns of PkNF YB/C genes in ABA treatment. CK: white space handling; +ABA: somatic embryos grown on mature medium supplemented with 80 μmol∙L^−1^ ABA; −ABA: somatic embryos grown on mature medium supplemented without ABA. The transcriptome expression data were normalized using CK as the control standard, while the data from other treatment groups were normalized using log_2_^FPKM (treatment)/FPKM (CK)^ as values.

As shown in Figure 9, *PkNF-YB 2* showed almost no expression in both the control and ABA treatments. Compared with the control group, the expression levels of *PkNF-YB 7*, *PkNF-YC 3*, and *4* increased and maintained high levels in the treatment with added ABA, while in the treatment without added ABA, the expression levels decreased in the second week of culture. However, the expression levels of *PkNF-YC 1*, *5*, and *PkNF-YB 4* showed a decreasing trend in both the treatment with and without ABA, but the decreasing trend was faster in the treatment without ABA, reaching almost undetectable levels in the second week. On the contrary, *PkNF YB 1* and *5* showed a rapid downward trend in the treatment with added ABA and dropped to almost undetectable levels in the second week of treatment. The expression levels of *PkNF-YB 3*, *6*, *PkNF-YC 2*, and *7* were higher in the control and non-ABA treated groups.

### 2.7. Subcellular Localization and Transcriptional Self-Activation Analysis of Transcription Factor

Based on the expression pattern of the *PkNF-YB/C* genes, all genes were selected for subsequent experiments, except for *PkNF-YB 2* and *PkNF-YC 6*, which were not expressed during the development of somatic and zygotic embryos. Instantaneous transformation was performed by injecting tobacco leaves into the lower epidermis to detect the subcellular localization of the NF-YB/C proteins.

**Figure 10:**
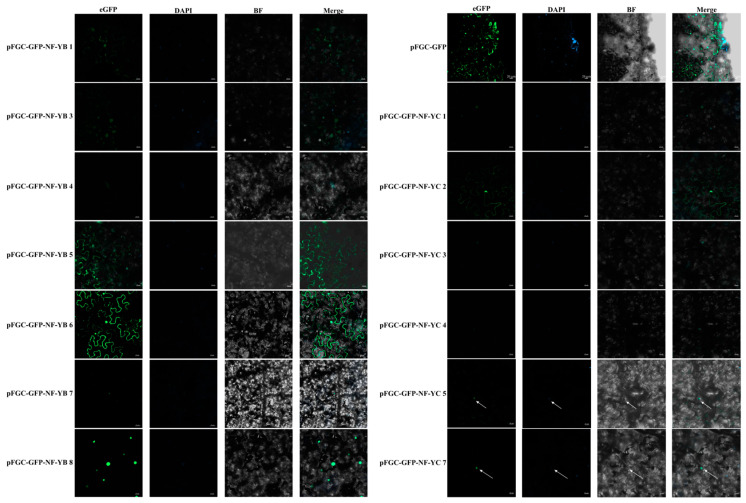
Subcellular localization of *PkNF-YB/C* proteins. DAPI: a nuclear staining dye; BF: bright field; merge: The merged images of BF, eGFP, and DAPI staining. White arrow indicates the location of the cell nucleus.

**Figure 11:**
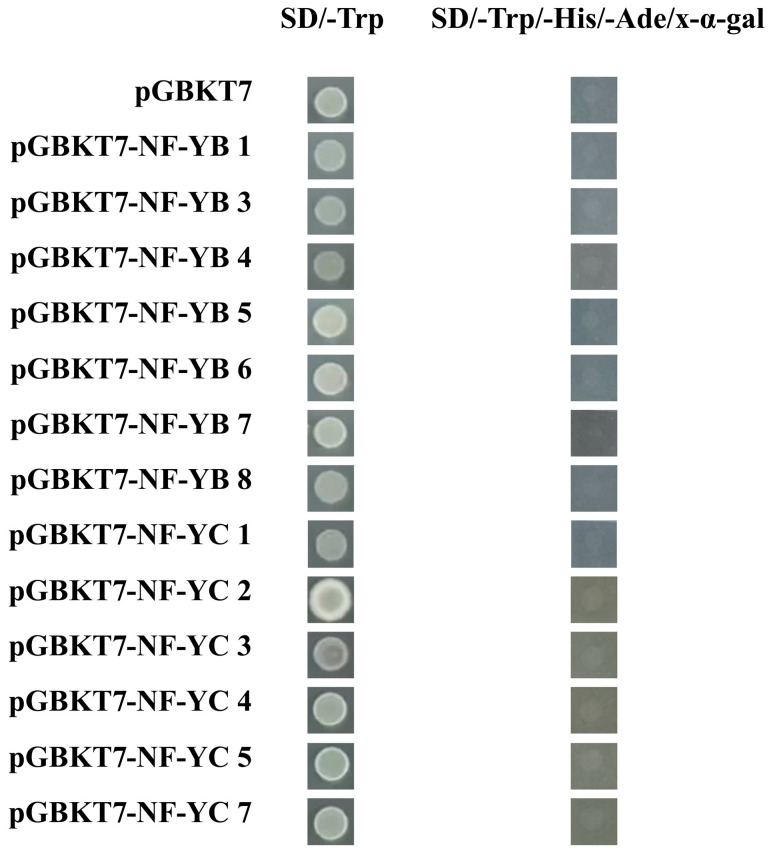
Analysis of the transcriptional activation activity of *PkNF-YB/C* proteins. pGBKT7: negative control.

As shown in Figure 10, similar to empty vector, PkNF-YB 1, 3, 5, and 6 were located in the nucleus and cell membrane, while PkNF-YB 4, 7, and 8 are co-located in the nucleus with DAPI staining. Among the PkNF-YC proteins, except for PkNF-YC2, which was located in the nucleus and nucleus, all other proteins were located in the nucleus. As shown in Figure 11, analyze the transcriptional activation activity of PkNF-YB/C proteins by using the yeast hybridization system (Y2H). The Y2H yeast cells corresponding to the PkNF-YB/C proteins fused with the GAL4 DNA-binding domain (GALDA4DB) can grow normally on SD/-Trp medium but cannot grow on SD/-Trp/-His/-Ade/x-α-gal medium. This is consistent with the growth status of the negative control, indicating that the PkNF-YB/C proteins do not possess transcriptional autoactivation activity.

### 2.8. Predicting Protein Interaction Networks for PkNF-YB and PkNF-YC Genes

**Figure 12:**
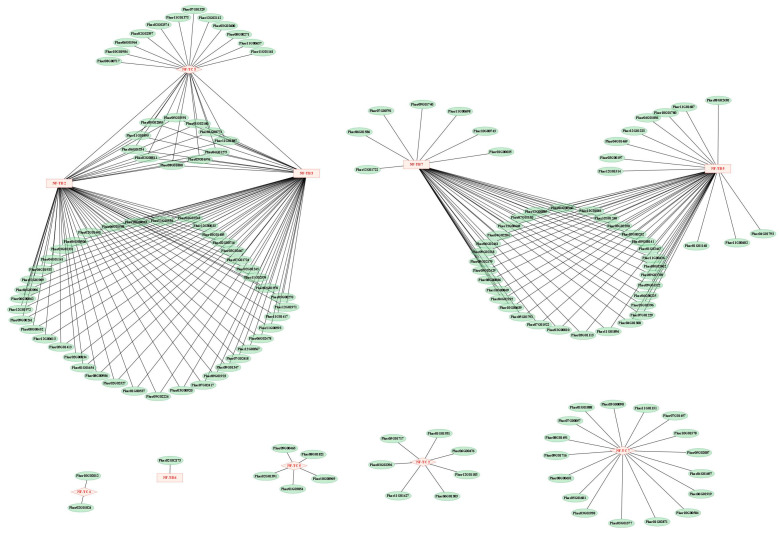
The protein interaction network of PkNF-YB and PkNF-YC. The red nodes represent the PkNF-YB and PkNF-YC proteins, while the green nodes represent the predicted interaction proteins. The connecting lines represent the interacting relationships.

We further identified putative interacting proteins for PkNF-YB/C (Figure 12). Among them, PkNF-YB 2, 3, 5, 6, and 7 predicted 54, 54, 43, 1, and 39 interacting proteins, respectively. PkNF-YC 2, 3, 4, 5, and 7 predicted 7, 25 (including PkNF-YB 2 and 3), 2, 5, and 17 interacting proteins, respectively. The description of the interacting proteins is shown in Table S7.

### 2.9. Screening and Mature Phenotype of PkNF-YB 7 Transgenic Cell Lines

*PkNF-YB 7* was named *PkLEC1* because it is homologous to LEC1 in Arabidopsis. To characterize its function during somatic embryogenesis, a plant overexpression vector was constructed and genetically transformed into *P. koraiensis* callus tissue to obtain multiple transgenic callus cell lines.

**Figure 13:**
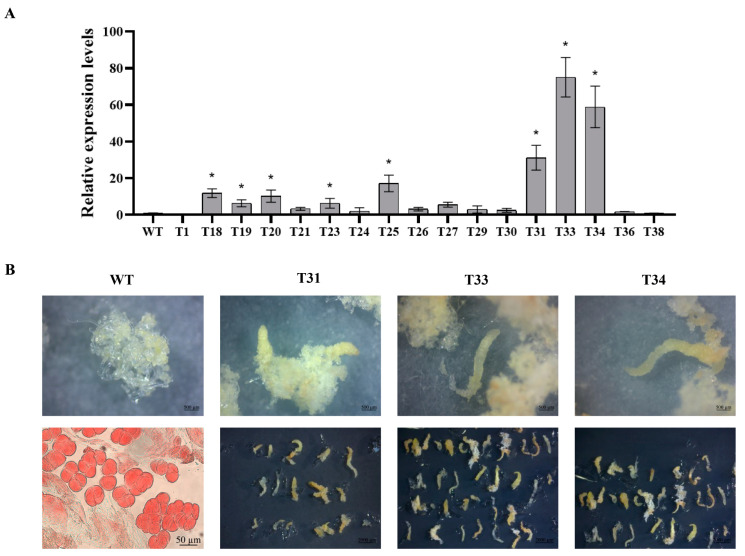
Identification and transgenic lines of PkLEC1 in *P. koraiensis*. qRTPCR detection of PkLEC1 transgenic lines (**A**). Pk18S was used as a control. Each error bar represents the standard deviation of three biological replicates. Asterisks indicate levels of significance (Dunnett’s test; *, *p* < 0.05). Mature phenotype of *PkNF-YB 7* transgenic cell lines (**B**). WT and *PkNF-YB 7* transgenic lines under 8-week mature culturation (T31, T33, and T34).

QRT-PCR was used to quantitatively analyze the expression levels of *PkLEC1* in these transgenic lines. Three transgenic strains (T31, T33, T34) were selected for subsequent experiments because their *PkLEC1* expression levels were higher than other strains (Figure 13A).

According to the method described by Peng et al. [61], the somatic embryogenesis was tested. The wild-type callus cells were previously induced from immature seeds by the research group and had completely lost their ability of somatic embryogenesis (L-SE) before genetic transformation began.

As shown in Figure 13B, there were no observable mid- or late-stage somatic embryos in the wild type cultured for 2 months. However, transgenic cell lines that have been cultured for 2 months have observable mid- or late-stage somatic embryos, some of which have even developed into cotyledons (Figure S1). The results indicate that overexpression of PkLEC1 enhances the somatic embryogenesis ability of L-SE to a certain extent.

## 3. Discussion

Previous studies have shown that NF-YB and NF-YC family transcription factors are involved in various biological processes in plants [13,23,28,62,63,64,65]. The *NF-YB/C* family has been characterized and identified in multiple species, such as *Arabidopsis* [66], rice [67], wheat [68], soybeans [69], and Populus [70]. However, there is limited research in gymnosperms, especially in *P. koraiensis*, which has not yet been characterized.

This article identifies the *NF-YB* and *NF-YC* gene families for the first time in *P. koraiensis*, with high economic and ecological value in the Northeast region in China. Gene family identification and phylogenetic analysis indicate that the NF-YB gene family of *P. koraiensis* includes eight gene members, and the *NF-YC* gene family includes seven members, which have close evolutionary relationships with the gene families of *P. taeda*, *P. koraiensis* s and *P. abies* (Figure 1). The motif analysis shows that NF-YBs have highly conserved HFM (H2B) motifs, while NF-YCs have highly conserved HFM (H2A) motifs (Figure 1). Collinearity analysis showed that four and four collinearity relationships were identified within the *NF-YB* and *NF-YC* families of *P. koraiensis* and *P. taeda*. This indicates that these two families are relatively conservative in their evolutionary relationship, and research on the NF-YB/C family in other gymnosperms is of great reference value in *P. koraiensis*. And Ka/Ks analysis showed that the repetitive gene pairs were mainly in purification selection (Ka/Ks < 1.0).

Research has shown that many plant transcription factor promoters have highly conserved *cis*-elements that play a crucial role in transcriptional regulatory signaling pathways when plants are subjected to both biological and abiotic stresses [71,72]. Therefore, the cis-acting elements in the promoter region of the *PkNF-YB/C* genes were analyzed, and it was found that each gene contains at least three elements related to hormones or biological stress. Thus, we speculate that *PkNF-YB/C* genes may also play an important role in plant hormone response and stress resistance. This provides important ideas for the subsequent study of gene function. This is also consistent with previous studies in other species [73,74].

Tissue expression specificity analysis showed that *PkNF-YB* and *PkNF-YC* members were differentially expressed in different tissue parts, indicating their involvement in different developmental processes. The differential functional roles of NF-YB/C family members in different stages of plant growth and development are explained, with less redundancy in co-expression, which is different from angiosperms. Subcellular localization analysis shows that both PkNF-YB and PkNF-YC proteins have nuclear localization, indicating that they are transcription factors. Transcription activation experiments have shown that both PkNF-YB and PkNF-YC do not possess transcriptional activation activity. Due to the fact that NF-YBs generally form heterodimers with NF-YC proteins, and then form oligomers with NF-YA proteins or other transcription factors to regulate downstream target genes. In contrast, most previously reported NF-YB and NF-YC transcription factors are exclusively localized in the nucleus. This may also be attributed to the scarcity of research on NF-YB and NF-YC.

Then, we further predicted the protein interaction network of PkNF-YB/C members. A large number of interacting genes were found in PkNF-YB 2, 3, 5, 7, PkNF-YC 3, and 7. The interaction genes between PkNF-YB 2 and PkNF-YB3 overlap, and both interact with PkNF-YC 3. In addition, PkNF-YB 5 and PkNF-YB 7 also share many common interacting genes. This further confirms that NF-YB/C family proteins function in the form of oligomers. Among them, PkNF YB2 and PkNF YB3, as well as PkNF YB5 and PkNF YB7, share multiple potential interacting proteins, indicating that they are functionally similar. And PkNF YC 2, 3, 4, 5, and 7 may be the more core genes in the NF-YC family, especially PkNF YC 3, because it has a close relationship with the NF-YB family and has multiple predicted potential interacting proteins. These genes that interact with multiple PkNF-YB/C members deserve our further attention and research.

The overexpression of *PkLEC1* can enhance the somatic embryogenesis ability of L-SE, extending callus that can only develop to the early stage of somatic embryogenesis to the middle or even late stage of somatic embryogenesis, and improving the quality of somatic embryogenesis. So, we speculate that the overexpression of *PkLEC1* can improve the quality of somatic embryogenesis in callus with poor or good somatic embryogenesis ability, consistent with studies on other species.

## 4. Materials and Methods

### 4.1. Plant Materials and Growth Conditions

The experimental material was the embryogenic callus of *P. koraiensis* preserved in our laboratory, which consisted of Modified Litvay medium (MLV) (Coolaber, Beijing, China) [61] supplemented with 0.5 mg·L^−1^ of 2,4-D (Phytotech, Kansas, America), 0.1 mg·L^−1^ of 6-BA (Phytotech, Kansas, America), 30 g·L^−1^ of sucrose, 0.5 g·L^−1^ of L-glutamine (Sinopharm Chemical, Shanghai, China), and 0.5 g·L^−1^ of CH (Sinopharm Chemical, Shanghai, China). The pH of the medium was then adjusted to 5.8 before sterilization. Embryonic callus (EC) was subcultured at intervals of 2 weeks to maintain and proliferate, with the culture placed in darkness at 24 ± 1°C. Embryogenic callus tissue cultured for 7 days was transferred to mature medium for cultivation. The mature medium was MLV with 80 μmol·L^−1^ of ABA, 1.2% gellan gum, and 0.2 mol·L^−1^ sucrose, pH 5.8. They were cultivated in the dark for 8 weeks to count the number of somatic embryos [61]. *Nicotiana tabacum* seedlings were propagated and planted in a mixture of turfy peat and sand (1:1 *v*/*v*) and grown under a 16 h/8 h day/night photoperiod at 25 ± 1°C.

### 4.2. Identification of PkNF-YB/C Family Genes in P. koraiensis and Physicochemical Property Analysis

In order to identify NF-YB and NF-YC proteins in *P. koraiensis*, according to the *P. koraiensis* protein database (not yet published) and Hidden Markov Model (HMM), File CBFD_NFYB_HMF (PF00808) was downloaded from the Pfam database (https://pfam.xfam.org/, accessed on 16 June 2023). Using the hmmsearch command in HMMER (v3.1) software, the *P. koraiensis* protein database was searched with PF00808.hmm file, and the identified sequences were integrated [75]. After screening and identification, 8 NF-YB amino acid sequences and 7 NF-YC amino acid sequences of *P. koraiensis* were obtained. The characteristics of these amino acid sequences, including predicted molecular weight, isoelectric point, number of amino acids, and grand average of hydropathicity (GRAVY) score were analyzed using the online ExPASy program (https://www.expasy.org/, accessed on 17 September 2024).

### 4.3. Multiple Sequence Alignment and Phylogenetic Analysis

The NF-YB and NF-YC amino acid sequences were aligned using MUSCLE and checked for the presence of the conserved HFM (H2B) and HFM (H2A) sites, respectively. The maximum likelihood method was used to construct phylogenetic trees (1000 bootstrap replications) using MEGA (accessed on 17 January 2025) [76].

### 4.4. Exon/Intron Structure and Conserved Motifs Analysis

The conserved domains of PkNF-YBs and PkNF-YCs were predicted and analyzed by the online tool MEME (https://meme-suite.org/meme/tools/meme, accessed on 19 November 2023) [77]. The Motif Number was set to 4, and the remaining parameters were set to Default. The gene structure information was extracted and visualized by TBTools v2.003 [78].

### 4.5. Chromosomal Location, Collinearity Analysis, and Ka/Ks Calculation

Extract the location information of NF-YB and NF-YC family members from the *P. koraiensis* gff file. TBtools was used to construct the circular chromosome distribution map and interspecies collinearity analysis of genes. We downloaded chromosome files and gff3 files of *Pinus tabulaeformis* and *Arabidopsis thaliana* from NCBI, and TBtools was used for multispecies collinearity alignment and visualization [78]. TBtools v2.102 was used to calculate the Ka and Ks values (accessed on 18 March 2024) (Table S1).

### 4.6. Cis-Element Analysis of the PkNF-YB/C Promoters

The upstream 2000 bp of transcriptional initiation of family members was defined as the promoter region of *PkNF-YB/C* genes for analysis. The elements in the promoter region were predicted using the PlantCARE (http://bioinformatics.psb.ugent.be/webtools/plantcare/html/, accessed on 12 November 2023) online website [79] and visualized with TBtools.

### 4.7. GO and KEGG Enrichment Analysis of PkNF-YB/C Genes

TBtools was used to visualize the GO enrichment and KEGG enrichment analysis of *PkNF-YB/C* genes.

### 4.8. Expression Patterns of PkNF-YB/C Genes

Based on the transcriptome data of roots, stems, leaves, seeds, embryonic callus, non-embryonic callus, somatic embryos, zygotic embryos from different tissue parts of *P. koraiensis* in our research, as well as transcriptome data of megasporophylls, microspores, flower buds, and ovules at different developmental stages, the gene expression levels of *PkNF-YB/C* genes were analyzed and gene expression calorimeter maps were drawn. The expression levels of *PkNF-YB/C* genes were analyzed based on transcriptome data from 80 μmol·L^−1^ ABA-treated callus of *P. koraiensis* in our research.

### 4.9. Cloning PkNF-YB/C Genes from P. koraiensis

The full *PkNF-YB/C* genes were amplified with gene-specific primers (Table S2) by using the published method, and then transformed into *Escherichia coli* cells (DH5α, ANGYUBIO) for validation by Sanger sequencing.

### 4.10. Transcriptional Repression in Yeast

The transcriptional activation of *PkNF-YB/C* genes and the GAL4BD/UAS/LacZ transient assays were performed in yeast cells. Yeast GaL4BD expression vectors were obtained from Clontech. The complete CDS sequences of *PkNF-YB/C* genes were amplified using specifically designed primers (Table S2). The sequences encoding *PkNF-YB/C* genes were amplified by PCR and cloned into *BamH* I sites of the pGBKT7 vector (Clontech). The amplified fragments were fused in-frame to the pGBKT7 vector to generate the pGBKT7-*PkNF-YB/Cs* construct. The pGBKT7-*PkNF-YB/Cs* and the pGBKT7 blank vector (as negative control) were transformed into Y2H yeast cells independently. The transformed Y2H yeast cells were plated onto SD/-Trp (growth control), SD/-Trp/-His/-Ade, and X-α-Gal media and incubated at 30 °C for three to five days to identify the transcriptional activation.

### 4.11. Subcellular Localization of PkNF-YB/C Genes

The full-length coding regions of *PkNF-YB/Cs* without termination codon were amplified using specific primers (Table S2) and then fused to the N-terminal of GFP driven by a CaMV 35S promoter in the pFGC-eGFP vector. The two fusion constructs (pFGC-eGFP and pFGC- *PkNF-YB/Cs*-eGFP) were injected into *Nicotiana tabacum* epidermal cells. The GFP fluorescent images were photographed with confocal microscopy (ZEISS LSM 800, Shanghai, China) at 48 h after injected.

### 4.12. Genetic Transformation of P. koraiensis Callus for Generating PkNF-YB 7 Transgenic Lines

The PkNF-YB7 was amplified with specific primers, and then inserted into the pCambia1301 vector at the position immediately downstream of the CaMV 35S promoter, and transferred into *Agrobacterium tumefaciens* GV3101 using the freeze–thaw method. We performed genetic transformation based on published patents [80]. Genomic DNA of all hygromycin-resistant shoots was amplified by regular PCR using the p1301 sequencing primers listed in Table S1 to verify whether *PkNF-YB 7* was integrated into the *P. koraiensis* genome. We performed real-time RT-qPCR on various transgenic cell lines, using the Pk18S gene from red pine as the internal reference gene. The forward primer sequence for detecting the *Pk18S* was 5′-GGGTGGTTTATGTTTG-3′, and the reverse primer sequence was 5′-GAAAGGGTTGAGGAAG-3. The forward primer sequence for detecting the *PkLEC1* was 5′-TGTCCGAGAGTGGCAGCC-3′, and the reverse primer sequence was 5′-GCATGGGTGGGCAGAACT-3′.

### 4.13. Observation of Phenotypic Characteristics of Transgenic Strains

Phenotypic differences between wild-type and transgenic callus lines during the proliferation and somatic embryo maturation stages were observed.

## 5. Conclusions

This article identifies the *NF-YB* and *NF-YC* gene families for the first time in *P. koraiensis* with high economic and ecological value in the Northeast region in China. Gene family identification and phylogenetic analysis indicate that the NF-YB gene family of *P. koraiensis* includes eight gene members, and the *NF-YC* gene family includes seven members, which have close evolutionary relationships with the gene families of *P. taeda*, *P. koraiensis* s and *P. abies* (Figure 1). The motif analysis shows that NF-YBs have highly conserved HFM (H2B) motifs, while NF-YCs have highly conserved HFM (H2A) motifs (Figure 1). Collinearity analysis showed that four and four collinearity relationships were identified within the *NF-YB* and *NF-YC* families of *P. koraiensis* and *P. taeda*. And Ka/Ks analysis showed that the repetitive gene pairs were mainly in purification selection (Ka/Ks < 1.0).

The *cis* elements in the promoter region of the *PkNF-YB/C* genes were analyzed, and it was found that each gene contains at least three elements related to hormones or biological stress. A tissue expression specificity analysis showed that *PkNF-YB* and *PkNF-YC* members were differentially expressed in different tissue parts, indicating their involvement in different developmental processes. Subcellular localization analysis shows that both PkNF-YB and PkNF-YC proteins have nuclear localization, indicating that they are transcription factors. Transcription activation experiments have shown that both PkNF-YB and PkNF-YC do not possess transcriptional activation activity. A large number of interacting genes were found in PkNF-YB 2, 3, 5, 7, PkNF-YC 3, and 7. The interacting genes between PkNF-YB 2 and PkNF-YB3 overlap, and both interact with PkNF-YC 3. In addition, PkNF-YB 5 and PkNF-YB 7 also share many common interacting genes.

The overexpression of *PkLEC1* can enhance the somatic embryogenesis ability of L-SE, extending callus that can only develop to the early stage of somatic embryogenesis to the middle or even late stage of somatic embryogenesis, and improving the quality of somatic embryogenesis.

## Figures and Tables

**Table 1:** Parameters for the ten identified PkNF-YB/Cs and deduced polypeptide sequences present in the *P. koraiensis* genome.

Gene Name	Locus Name	Amino Acid No.	Molecular Weight (Da)	Isoelectric Points	GRAVY	Chromosome Location
*PkNF-YB1*	Pkor01G01777	225	24,436.99	6.08	−0.866	Chr01:1563581603..1563582280
*PkNF-YB2*	Pkor02G00711	220	23,832.19	7.76	−0.931	Chr02:805110131..805110793
*PkNF-YB3*	Pkor03G01857	194	21,331.17	6.65	−0.646	Chr03:1580969493..1580970077
*PkNF-YB4*	Pkor06G00913	189	20,187.22	6.75	−0.877	Chr06:823725832..823726401
*PkNF-YB5*	Pkor08G00867	163	18,176.36	5.67	−0.798	Chr08:877008868..877021563
*PkNF-YB6*	Pkor08G02153	78	8531.37	4.96	−0.738	Chr08:1957384587..1957384823
*PkNF-YB7*	Pkor10G01254	184	20,743.25	5.15	−0.694	Chr10:1036699488..1036700042
*PkNF-YB8*	Pkor11G01542	177	20,291.51	5.36	−0.801	Chr11:1222883089..1222883622
*PkNF-YC1*	Pkor02G01005	202	22,438.70	6.20	−0.409	Chr02:1063370683..1063371291
*PkNF-YC2*	Pkor06G01119	272	30,067.30	5.96	−0.440	Chr06:993282521..993283339
*PkNF-YC3*	Pkor08G01708	236	27,453.39	6.13	−0.687	Chr08:1604161452..1604167962
*PkNF-YC4*	Pkor09G00165	265	30,137.92	6.89	−0.634	Chr09:166805975..166806772
*PkNF-YC5*	Pkor09G00166	261	29,105.97	8.90	−0.452	Chr09:167008318..167009103
*PkNF-YC6*	Pkor09G00167	250	27,833.70	8.42	−0.301	Chr09:168737804..168738556
*PkNF-YC7*	Pkor10G02232	311	35,237.53	6.49	−0.917	Chr10:1969147323..1969148258

## Data Availability

The genome and transcriptome provided in this study are not yet published; therefore, login numbers cannot be provided. We apologize for any inconvenience caused. The datasets supporting the conclusions of this article are included within the article and its Supplementary Files.

## Supplementary Materials

The following supporting information can be downloaded at: https://www.mdpi.com/article/10.3390/plants14030438/s1, Table S1: The Ka/Ks ratios of duplication for PkNF-YB/Cs; Table S2: The prime list of PkNF-YB/Cs; Table S3: The prime list of PkNF-YB/Cs for analysis of transcriptional activation activity and subcellular localization; Table S4: Motif sequences of PkNF-YBs predicted in *P. koraiensis* using MEME tools; Table S5: Motif sequences of PkNF-YCs predicted in *P. koraiensis* using MEME tools; Table S6: The Synlinea of PkNF-YB/Cs between *Pinus tabulaeformis* and *Pinus koraiensis*; Table S7: Analysis of cis-acting elements in the promoters of PkNF-YB/Cs; Table S8: The annotation of the interacting proteins; Table S9: The node ID of the interacting proteins; Figure S1: Early cotyledon-type embryo from transgenic somatic embryo; Figure S2: GFP fluorescence observation of wild-type and transgenic embryonic callus tissues.
