# Supplementary material for: Genome-Wide Identification and Expression Pattern Analysis of Nuclear Factor Y B/C Genes in Pinus koraiensis, and Functional Identification of LEAFY COTYLEDON 1
Source: Plants (Basel). 2025 Feb 2;14(3):438. doi: 10.3390/plants14030438 (PMC11819940; doi:10.3390/plants14030438)
Supplement: Supplementary file 1 [file plants-14-00438-s001.zip › plants-3410987-supplementary.pdf]

Table S1. The Ka/Ks ratios of duplication for *PkNF-YB/C*s.

Seq_1 NAME	Seq_1	Seq_2 NAME	Seq_2	Ka	Ks	Ka_Ks	Effective Len
PkNF-YB 1	Pkor01G01777.1	PkNF-YB 2	Pkor02G00711.1	0.264887	3.056056	0.086676	621
PkNF-YB 1	Pkor01G01777.1	PkNF-YB 3	Pkor03G01857.1	0.305802	2.401453	0.127341	546
PkNF-YB 1	Pkor01G01777.1	PkNF-YB 5	Pkor08G00867.1	0.270271	2.308187	0.117092	480
PkNF-YB 1	Pkor01G01777.1	PkNF-YB 6	Pkor08G02153.1	0.307395	2.545204	0.120774	450
PkNF-YB 2	Pkor02G00711.1	PkNF-YB 3	Pkor03G01857.1	0.365392	2.386043	0.153137	561
PkNF-YB 2	Pkor02G00711.1	PkNF-YB 6	Pkor08G02153.1	0.29254	2.25142	0.129936	459
PkNF-YB 2	Pkor02G00711.1	PkNF-YB 8	Pkor11G01542.1	0.528783	2.270488	0.232894	531
PkNF-YB 3	Pkor03G01857.1	PkNF-YB 5	Pkor08G00867.1	0.346292	2.083319	0.166222	429
PkNF-YB 3	Pkor03G01857.1	PkNF-YB 6	Pkor08G02153.1	0.391495	4.408302	0.088809	456
PkNF-YB 5	Pkor08G00867.1	PkNF-YB 6	Pkor08G02153.1	0.179877	1.811446	0.0993	438
PkNF-YB 7	Pkor10G01254.1	PkNF-YB 8	Pkor11G01542.1	0.113325	2.644588	0.042852	531
PkNF-YC 1	Pkor02G01005.1	PkNF-YC 4	Pkor09G00165.1	0.311172	1.897691	0.163974	597
PkNF-YC 1	Pkor02G01005.1	PkNF-YC 7	Pkor10G02232.1	0.323403	3.05177	0.105972	576
PkNF-YC 2	Pkor06G01119.1	PkNF-YC 4	Pkor09G00165.1	0.25703	3.48155	0.073826	750
PkNF-YC 4	Pkor09G00165.1	PkNF-YC 7	Pkor10G02232.1	0.431527	3.447166	0.125183	717

Table S2. The Prime List of *PkNF-YB/Cs*

Primes Name	Sequence
PkNF-YB 1-F	5'-ATGGCAGAGAACTATGGCAG-3'
PkNF-YB 1-R	5'-CTACCATTGGCCTCTGGG-3'
PkNF-YB 3-F	5'-ATGGCGTCCACTCGGCAT-3'
PkNF-YB 3-R	5'-CTACCACTGCCCTCTAGGTTGAT-3'
PkNF-YB 4-F	5'-ATGGCTTACAACCGCCAC-3'
PkNF-YB 4-R	5'-TCACCTTCCATGCCCGAA-3'
PkNF-YB 5-F	5'-ATGGCGGAAGCCAGCAGC-3'
PkNF-YB 5-R	5'-TCATGACAGATCATTGCCCTGC-3'
PkNF-YB 6-F	5'-ATGGCAGACTTAGCGAGCTC-3'
PkNF-YB 6-R	5'-TTACTCGCTGGTAATGAAACTGA-3'
PkNF-YB 7-F	5'-ATGTTTGCAGGTATGATGTCC-3'
PkNF-YB 7-R	5'-TCACTTATACTGAGCATAGGGATC-3'
PkNF-YB 8-F	5'-ATGGCAGAAGATGCAAGCC-3'
PkNF-YB 8-R	5'-TCACTTATATGGAGCATATGGATCA-3'
PkNF-YC 1-F	5'-ATGGATCAGCAGCAGCCC-3'
PkNF-YC 1-R	5'-TCAACTGCCGCCATGAGG-3'
PkNF-YC 2-F	5'-ATGGAGCAGCAAGCCCCA-3'
PkNF-YC 2-R	5'-CTAACTGCTAGAGGATGGGCC-3'
PkNF-YC 3-F	5'-ATGGAAGGCAAGTACCCACAT-3'
PkNF-YC 3-R	5'-TCATTGCTGTGGAGGTTGGTA-3'
PkNF-YC 4-F	5'-ATGGATAAGCAATCCACAATG-3'
PkNF-YC 4-R	5'-CTATCCAGAGTTTGGTGATTTTG-3'
PkNF-YC 5-F	5'-ATGGAACAGAAATCCTCGAG-3'
PkNF-YC 5-R	5'-TTAACCAGAATTGGGTGACT-3'
PkNF-YC 7-F	5'-ATGGACCACCACAACCACC-3'
PkNF-YC 7-R	5'-TCAGTTGGCAGAACGAGG-3'

Table S3. The Prime List of *PkNF-YB/Cs* for analysis of transcriptional activation activity and subcellular localization

Primes Name	Sequence
NF-YB 1-BD-F	5'-GCATATGGCCATGGAGGCCGAATTCATGGCAGAGAACTATGGCAGCCCGGATAG-3'
NF-YB 1-BD-R	5'-CGGCCGCTGCAGGTCGACGGATCCCTACCATTGGCCTCTGGGGG-3'
NF-YB 3-BD-F	5'-GCATATGGCCATGGAGGCCGAATTCATGGCGTCCACTCGGCATCAGCG-3'
NF-YB 3-BD-R	5'-CGGCCGCTGCAGGTCGACGGATCCCTACCACTGCCCTCTAGGTTGAT-3'
NF-YB 4-BD-F	5'-GCATATGGCCATGGAGGCCGAATTCATGGCTTACAACCGCCACCAT-3'
NF-YB 4-BD-R	5'-CGGCCGCTGCAGGTCGACGGATCCTCACCTTCCATGCCCGAAAG-3'
NF-YB 5-BD-F	5'-GCATATGGCCATGGAGGCCGAATTCATGGCGGAAGCCAGCAGCCCGGG-3'
NF-YB 5-BD-R	5'-CGGCCGCTGCAGGTCGACGGATCCTCATGACAGATCATTGCCCTGCA-3'
NF-YB 6-BD-F	5'-GCATATGGCCATGGAGGCCGAATTCATGGCAGACTTAGCGAGCTCCGC-3'
NF-YB 6-BD-R	5'-CGGCCGCTGCAGGTCGACGGATCCTTACTCGCTGGTAATGAAACTGA-3'
NF-YB 7-BD-F	5'-GCATATGGCCATGGAGGCCGAATTCATGTTTGCAGGTATGATGTC-3'
NF-YB 7-BD-R	5'-CGGCCGCTGCAGGTCGACGGATCCTCACTTATACTGAGCATAGG-3'
NF-YB 8-BD-F	5'-GCATATGGCCATGGAGGCCGAATTCATGTTTGCAGGTATGATGTC-3'
NF-YB 8-BD-R	5'-CGGCCGCTGCAGGTCGACGGATCCTCACTTATATGGAGCATATG-3'
NF-YC 1-BD-F	5'-GCATATGGCCATGGAGGCCGAATTCATGGATCAGCAGCAGCCCACAAT-3'
NF-YC 1-BD-R	5'-CGGCCGCTGCAGGTCGACGGATCCTCAACTGCCGCCATGAGGAGGAG-3'
NF-YC 2-BD-F	5'-GCATATGGCCATGGAGGCCGAATTCATGGAGCAGCAAGCCCCAGGCCA-3'
NF-YC 2-BD-R	5'-CGGCCGCTGCAGGTCGACGGATCCCTAACTGCTAGAGGATGGGCC-3'
NF-YC 3-BD-F	5'-GCATATGGCCATGGAGGCCGAATTCATGGAAGGCAAGTACCCACATCT-3'
NF-YC 3-BD-R	5'-CGGCCGCTGCAGGTCGACGGATCCTCATTGCTGTGGAGGTTGGTACA-3'
NF-YC 4-BD-F	5'-GCATATGGCCATGGAGGCCGAATTCATGGATAAGCAATCCACAAT-3'
NF-YC 4-BD-R	5'-CGGCCGCTGCAGGTCGACGGATCCCTATCCAGAGTTTGGTGATT-3'
NF-YC 5-BD-F	5'-GCATATGGCCATGGAGGCCGAATTCATGGAACAGAAATCCTCGAG-3'
NF-YC 5-BD-R	5'-CGGCCGCTGCAGGTCGACGGATCCTTAACCAGAATTGGGTGACT-3'
NF-YC 7-BD-F	5'-GCATATGGCCATGGAGGCCGAATTCATGGACCACCACAACCACCA-3'
NF-YC 7-BD-R	5'-CGGCCGCTGCAGGTCGACGGATCCTCAGTTGGCAGAACGAGGGG-3'
NF-YB 1-pFGC-F	5'-GCATGGACGAGCTGTACAAGGGATCCATGGCAGAGAACTATGGCAGCCCGGATAG-3'
NF-YB 1-pFGC-R	5'-ATTAACTCTCTAGACTCACCTAGGATCCCTACCATTGGCCTCTGGGGG-3'
NF-YB 3-pFGC-F	5'-GCATGGACGAGCTGTACAAGGGATCCATGGCGTCCACTCGGCATCAGCG-3'
NF-YB 3-pFGC-R	5'-ATTAACTCTCTAGACTCACCTAGGATCCCTACCACTGCCCTCTAGGTTGAT-3'
NF-YB 4-pFGC-F	5'-GCATGGACGAGCTGTACAAGGGATCCATGGCTTACAACCGCCACCAT-3'

NF-YB 4-pFGC-R	5'-ATTAACTCTCTAGACTCACCTAGGATCCTCACCTTCCATGCCCGAAAG-3'
NF-YB 5-pFGC-F	5'-GCATGGACGAGCTGTACAAGGGATCCATGGCGGAAGCCAGCAGCCCGGG-3'
NF-YB 5-pFGC-R	5'-ATTAACTCTCTAGACTCACCTAGGATCCTCATGACAGATCATTGCCCTGCA-3'
NF-YB 6-pFGC-F	5'-GCATGGACGAGCTGTACAAGGGATCCATGGCAGACTTAGCGAGCTC-3'
NF-YB 6-pFGC-R	5'-ATTAACTCTCTAGACTCACCTAGGATCCTTACTCGCTGGTAATGAAACTGA-3'
NF-YB 7-pFGC-F	5'-GCATGGACGAGCTGTACAAGGGATCCATGTTTGCAGGTATGATGTCC-3'
NF-YB 7-pFGC-R	5'-ATTAACTCTCTAGACTCACCTAGGATCCTCACTTATACTGAGCATAGGGATC-3'
NF-YB 8-pFGC-F	5'-GCATGGACGAGCTGTACAAGGGATCCATGGCAGAAGATGCAAGCC-3'
NF-YB 8-pFGC-R	5'-ATTAACTCTCTAGACTCACCTAGGATCCTCACTTATATGGAGCATATGGATCA-3'
NF-YC 1-pFGC-F	5'-GCATGGACGAGCTGTACAAGGGATCCATGGATCAGCAGCAGCCC-3'
NF-YC 1-pFGC-R	5'-ATTAACTCTCTAGACTCACCTAGGATCCTCAACTGCCGCCATGAGG-3'
NF-YC 2-pFGC-F	5'-GCATGGACGAGCTGTACAAGGGATCCATGGAGCAGCAAGCCCCA-3'
NF-YC 2-pFGC-R	5'-ATTAACTCTCTAGACTCACCTAGGATCCCTAACTGCTAGAGGATGGGCC-3'
NF-YC 3-pFGC-F	5'-GCATGGACGAGCTGTACAAGGGATCCATGGAAGGCAAGTACCCACAT-3'
NF-YC 3-pFGC-R	5'-ATTAACTCTCTAGACTCACCTAGGATCCTCATTGCTGTGGAGGTTGGTA-3'
NF-YC 4-pFGC-F	5'-GCATGGACGAGCTGTACAAGGGATCCATGGATAAGCAATCCACAAT-3'
NF-YC 4-pFGC-R	5'-ATTAACTCTCTAGACTCACCTAGGATCCCTATCCAGAGTTTGGTGATT-3'
NF-YC 5-pFGC-F	5'-GCATGGACGAGCTGTACAAGGGATCCATGGAACAGAAATCCTCGAG-3'
NF-YC 5-pFGC-R	5'-ATTAACTCTCTAGACTCACCTAGGATCCTTAACCAGAATTGGGTGACT-3'
NF-YC 7-pFGC-F	5'-GCATGGACGAGCTGTACAAGGGATCCATGGACCACCACAACCACCA-3'
NF-YC 7-pFGC-R	5'-ATTAACTCTCTAGACTCACCTAGGATCCTCAGTTGGCAGAACGAGGGG-3'

Table S4. Motif sequences of PkNF-YBs predicted in *P. koraiensis* using MEME tools.

Motif	Width	E-value	Best possible match
1	60	1.40E-302	KETVQECVSEFISFITGEASDKCQKEKRKTINGDDLLWAMTTLGFEDYVEPLKIYLQKYR
2	31	2.20E-130	VREQDRFLPIANVGRIMKKALPANAKISKDA
3	11	7.50E-04	ELEGEKKGMAK
4	11	1.10E-03	DSSPRSEDESG

Table S5. Motif sequences of PkNF-YCs predicted in *P. koraiensis* using MEME tools.

Motif	Width	E-value	Best possible match
1	60	1.10E-273	HSLPLARIKKIMKADEDVRMISAEAPVVFAKACEMFILELTLRSWIHTEENKRRTLQKND
2	29	7.60E-89	IAAAITRTDIFDFLVDIVPRDELKEEGLG
3	31	4.20E-22	ANSFPYYYLPNQHSAPHGVIVGKPMDPAIYM
4	15	3.60E-18	QQLZIFWGNQMQEIE
5	24	2.10E-03	VGHMQNMLQRGYMNTDKSKSPNSG
6	11	1.20E-03	SVGSSSANAGG

Table S6. The Synlinear of NF-YB/Cs between *Pinus tabulaeformis* and *Pinus koraiensis*

P.koraiensis IDs				*P.tabulaeformis* IDs	
Chr02	Pkor02G00711.1	PkNF-YB 2	Collinearity to	Chr05	Pt5G11130.1
Chr02	Pkor02G01005.1	PkNF-YC 1	Collinearity to	Chr05	Pt5G04980.1
Chr06	Pkor06G00913.1	PkNF-YB 4	Collinearity to	Chr07	Pt7G10670.1
Chr08	Pkor08G02153.1	PkNF-YB 6	Collinearity to	Chr01	Pt1G51250.13
Chr08	Pkor08G01708.1	PkNF-YC 3	Collinearity to	Chr01	Pt1G63260.7
Chr08	Pkor08G00867.1	PkNF-YB 5	Collinearity to	Chr03	Pt3G66740.1
Chr09	Pkor09G00165.1	PkNF-YC 4	Collinearity to	Chr09	Pt9G28580.1
Chr10	Pkor10G02232.1	PkNF-YC 7	Collinearity to	Chr10	PtXG28240.1

Table S7. Analysis of *cis*-acting elements in the promoters of *PkNF-YB/Cs*

Genes	Start	Width	Element
PkNF-YB 1	1458	12	abscisic acid responsiveness
PkNF-YB 1	1463	6	abscisic acid responsiveness
PkNF-YB 1	1465	4	abscisic acid responsiveness
PkNF-YB 1	1495	10	abscisic acid responsiveness
PkNF-YB 1	1499	4	abscisic acid responsiveness
PkNF-YB 1	1597	6	abscisic acid responsiveness
PkNF-YB 1	1599	4	abscisic acid responsiveness
PkNF-YB 1	1740	12	cell cycle regulation
PkNF-YB 1	302	10	differentiation of the palisade mesophyll cells
PkNF-YB 1	912	6	drought responsiveness
PkNF-YB 1	1157	8	endosperm expression element
PkNF-YB 1	1207	8	endosperm expression element
PkNF-YB 1	1492	12	light responsiveness
PkNF-YB 1	552	6	light responsiveness
PkNF-YB 1	1454	18	light responsiveness
PkNF-YB 1	1458	12	light responsiveness
PkNF-YB 1	1463	6	light responsiveness
PkNF-YB 1	1497	6	light responsiveness
PkNF-YB 1	1593	12	light responsiveness
PkNF-YB 1	1597	6	light responsiveness
PkNF-YB 1	1463	6	light responsiveness
PkNF-YB 1	1597	6	light responsiveness
PkNF-YB 1	1328	6	light responsiveness
PkNF-YB 1	1696	6	light responsiveness
PkNF-YB 1	292	12	light responsiveness
PkNF-YB 1	458	6	light responsiveness
PkNF-YB 1	1971	8	light responsiveness
PkNF-YB 1	1753	10	light responsiveness
PkNF-YB 1	1635	6	low-temperature responsiveness
PkNF-YB 1	1911	6	low-temperature responsiveness
PkNF-YB 1	1539	4	MeJA responsiveness

PkNF-YB 1	1580	4	MeJA responsiveness
PkNF-YB 1	1611	4	MeJA responsiveness
PkNF-YB 1	1539	4	MeJA responsiveness
PkNF-YB 1	1580	4	MeJA responsiveness
PkNF-YB 1	1611	4	MeJA responsiveness
PkNF-YB 2	834	4	abscisic acid responsiveness
PkNF-YB 2	1981	10	auxin responsiveness
PkNF-YB 2	1979	6	drought responsiveness
PkNF-YB 2	679	8	gibberellin responsiveness
PkNF-YB 2	833	6	light responsiveness
PkNF-YB 2	589	8	light responsiveness
PkNF-YB 2	18	6	light responsiveness
PkNF-YB 2	367	6	light responsiveness
PkNF-YB 2	1918	12	light responsiveness
PkNF-YB 2	1923	12	light responsiveness
PkNF-YB 2	1045	12	light responsiveness
PkNF-YB 2	1784	8	light responsiveness
PkNF-YB 2	561	20	light responsiveness
PkNF-YB 2	1448	20	light responsiveness
PkNF-YB 2	625	6	light responsiveness
PkNF-YB 2	632	6	light responsiveness
PkNF-YB 2	673	6	light responsiveness
PkNF-YB 2	281	6	light responsiveness
PkNF-YB 2	537	12	light responsiveness
PkNF-YB 2	1553	4	MeJA responsiveness
PkNF-YB 2	1715	4	MeJA responsiveness
PkNF-YB 2	1977	4	MeJA responsiveness
PkNF-YB 2	1984	4	MeJA responsiveness
PkNF-YB 2	1553	4	MeJA responsiveness
PkNF-YB 2	1715	4	MeJA responsiveness
PkNF-YB 2	1977	4	MeJA responsiveness
PkNF-YB 2	1984	4	MeJA responsiveness
PkNF-YB 2	509	6	meristem expression

PkNF-YB 2	1965	12	wound responsiveness
PkNF-YB 2	1970	12	zein metabolism regulation
PkNF-YB 3	413	6	auxin responsiveness
PkNF-YB 3	1967	6	drought responsiveness
PkNF-YB 3	217	6	light responsiveness
PkNF-YB 3	708	6	light responsiveness
PkNF-YB 3	858	8	light responsiveness
PkNF-YB 3	860	6	light responsiveness
PkNF-YB 3	1939	8	light responsiveness
PkNF-YB 3	173	6	light responsiveness
PkNF-YB 3	361	6	light responsiveness
PkNF-YB 3	985	6	light responsiveness
PkNF-YB 3	1083	6	light responsiveness
PkNF-YB 3	1129	6	light responsiveness
PkNF-YB 3	1200	6	light responsiveness
PkNF-YB 3	1246	6	light responsiveness
PkNF-YB 3	1295	6	light responsiveness
PkNF-YB 3	1341	6	light responsiveness
PkNF-YB 3	1424	6	light responsiveness
PkNF-YB 3	1804	12	salicylic acid responsiveness
PkNF-YB 4	868	4	abscisic acid responsiveness
PkNF-YB 4	1487	8	gibberellin responsiveness
PkNF-YB 4	586	8	gibberellin responsiveness
PkNF-YB 4	866	6	light responsiveness
PkNF-YB 4	1410	6	light responsiveness
PkNF-YB 4	1776	6	light responsiveness
PkNF-YB 4	1272	8	light responsiveness
PkNF-YB 4	515	6	light responsiveness
PkNF-YB 4	464	12	light responsiveness
PkNF-YB 4	1669	4	MeJA responsiveness
PkNF-YB 4	1669	4	MeJA responsiveness
PkNF-YB 4	1697	6	meristem expression
PkNF-YB 4	485	10	seed-specific regulation

PkNF-YB 4	702	12	zein metabolism regulation
PkNF-YB 5	868	4	abscisic acid responsiveness
PkNF-YB 5	1973	8	gibberellin responsiveness
PkNF-YB 5	1352	8	gibberellin responsiveness
PkNF-YB 5	1487	8	gibberellin responsiveness
PkNF-YB 5	586	8	gibberellin responsiveness
PkNF-YB 5	18	6	light responsiveness
PkNF-YB 5	1454	6	light responsiveness
PkNF-YB 5	1088	12	light responsiveness
PkNF-YB 5	1620	12	light responsiveness
PkNF-YB 5	1394	14	light responsiveness
PkNF-YB 5	866	6	light responsiveness
PkNF-YB 5	1410	6	light responsiveness
PkNF-YB 5	1776	6	light responsiveness
PkNF-YB 5	1272	8	light responsiveness
PkNF-YB 5	515	6	light responsiveness
PkNF-YB 5	464	12	light responsiveness
PkNF-YB 5	1494	4	MeJA responsiveness
PkNF-YB 5	1916	4	MeJA responsiveness
PkNF-YB 5	1494	4	MeJA responsiveness
PkNF-YB 5	1916	4	MeJA responsiveness
PkNF-YB 5	1669	4	MeJA responsiveness
PkNF-YB 5	1669	4	MeJA responsiveness
PkNF-YB 5	1697	6	meristem expression
PkNF-YB 5	485	10	seed-specific regulation
PkNF-YB 5	702	12	zein metabolism regulation
PkNF-YB 6	1675	12	abscisic acid responsiveness
PkNF-YB 6	1680	6	abscisic acid responsiveness
PkNF-YB 6	1682	4	abscisic acid responsiveness
PkNF-YB 6	1880	12	defense and stress responsiveness
PkNF-YB 6	30	6	drought responsiveness
PkNF-YB 6	248	6	drought responsiveness
PkNF-YB 6	982	8	gibberellin responsiveness

PkNF-YB 6	1117	8	gibberellin responsiveness
PkNF-YB 6	1396	12	light responsiveness
PkNF-YB 6	1675	12	light responsiveness
PkNF-YB 6	1680	6	light responsiveness
PkNF-YB 6	1680	6	light responsiveness
PkNF-YB 6	1945	6	light responsiveness
PkNF-YB 6	524	6	light responsiveness
PkNF-YB 6	534	6	light responsiveness
PkNF-YB 6	1887	6	light responsiveness
PkNF-YB 6	1119	14	light responsiveness
PkNF-YB 6	1167	10	light responsiveness
PkNF-YB 6	504	4	MeJA responsiveness
PkNF-YB 6	1343	4	MeJA responsiveness
PkNF-YB 6	504	4	MeJA responsiveness
PkNF-YB 6	1343	4	MeJA responsiveness
PkNF-YB 7	1675	12	abscisic acid responsiveness
PkNF-YB 7	1680	6	abscisic acid responsiveness
PkNF-YB 7	1682	4	abscisic acid responsiveness
PkNF-YB 7	653	8	auxin responsiveness
PkNF-YB 7	1880	12	defense and stress responsiveness
PkNF-YB 7	183	6	drought responsiveness
PkNF-YB 7	781	6	drought responsiveness
PkNF-YB 7	30	6	drought responsiveness
PkNF-YB 7	248	6	drought responsiveness
PkNF-YB 7	1286	8	gibberellin responsiveness
PkNF-YB 7	982	8	gibberellin responsiveness
PkNF-YB 7	1117	8	gibberellin responsiveness
PkNF-YB 7	908	6	light responsiveness
PkNF-YB 7	1145	8	light responsiveness
PkNF-YB 7	1256	8	light responsiveness
PkNF-YB 7	862	12	light responsiveness
PkNF-YB 7	281	12	light responsiveness
PkNF-YB 7	275	6	light responsiveness

PkNF-YB 7	1170	8	light responsiveness
PkNF-YB 7	1396	12	light responsiveness
PkNF-YB 7	1675	12	light responsiveness
PkNF-YB 7	1680	6	light responsiveness
PkNF-YB 7	1680	6	light responsiveness
PkNF-YB 7	1945	6	light responsiveness
PkNF-YB 7	524	6	light responsiveness
PkNF-YB 7	534	6	light responsiveness
PkNF-YB 7	1887	6	light responsiveness
PkNF-YB 7	1119	14	light responsiveness
PkNF-YB 7	1167	10	light responsiveness
PkNF-YB 7	504	4	MeJA responsiveness
PkNF-YB 7	1343	4	MeJA responsiveness
PkNF-YB 7	504	4	MeJA responsiveness
PkNF-YB 7	1343	4	MeJA responsiveness
PkNF-YB 8	1265	4	abscisic acid responsiveness
PkNF-YB 8	854	8	gibberellin responsiveness
PkNF-YB 8	1919	8	gibberellin responsiveness
PkNF-YB 8	446	8	gibberellin responsiveness
PkNF-YB 8	1259	12	light responsiveness
PkNF-YB 8	1263	6	light responsiveness
PkNF-YB 8	49	14	light responsiveness
PkNF-YB 8	490	6	light responsiveness
PkNF-YB 8	803	6	light responsiveness
PkNF-YB 8	186	8	light responsiveness
PkNF-YB 8	1189	6	light responsiveness
PkNF-YB 8	506	12	light responsiveness
PkNF-YB 8	62	8	light responsiveness
PkNF-YB 8	464	20	light responsiveness
PkNF-YB 8	543	10	light responsiveness
PkNF-YB 8	1284	10	light responsiveness
PkNF-YB 8	1414	10	light responsiveness
PkNF-YB 8	1150	6	low-temperature responsiveness

PkNF-YB 8	1155	12	salicylic acid responsiveness
PkNF-YB 8	1459	12	zein metabolism regulation
PkNF-YC 1	323	4	anaerobic induction element
PkNF-YC 1	741	4	anaerobic induction element
PkNF-YC 1	1158	6	anaerobic induction element
PkNF-YC 1	1136	4	auxin responsiveness
PkNF-YC 1	739	6	gibberellin responsiveness
PkNF-YC 1	974	6	light responsiveness
PkNF-YC 1	546	6	light responsiveness
PkNF-YC 1	1768	10	light responsiveness
PkNF-YC 1	323	4	light responsiveness
PkNF-YC 1	797	8	light responsiveness
PkNF-YC 1	464	6	light responsiveness
PkNF-YC 1	1824	6	light responsiveness
PkNF-YC 1	176	6	light responsiveness
PkNF-YC 1	933	6	light responsiveness
PkNF-YC 1	1325	6	light responsiveness
PkNF-YC 1	1351	6	light responsiveness
PkNF-YC 1	1564	6	light responsiveness
PkNF-YC 1	52	12	MeJA responsiveness
PkNF-YC 1	519	4	MeJA responsiveness
PkNF-YC 1	1136	4	MeJA responsiveness
PkNF-YC 1	333	6	MeJA responsiveness
PkNF-YC 1	182	6	meristem expression
PkNF-YC 1	547	4	meristem expression
PkNF-YC 1	519	4	wound responsiveness
PkNF-YC 1	974	12	zein metabolism regulation
PkNF-YC 2	1983	6	abscisic acid responsiveness
PkNF-YC 2	1062	8	drought responsiveness
PkNF-YC 2	758	6	drought responsiveness
PkNF-YC 2	282	6	light responsiveness
PkNF-YC 2	1126	6	light responsiveness
PkNF-YC 2	1129	12	light responsiveness

PkNF-YC 2	1387	8	light responsiveness
PkNF-YC 2	1067	8	light responsiveness
PkNF-YC 2	1983	6	light responsiveness
PkNF-YC 2	1985	4	light responsiveness
PkNF-YC 2	153	6	light responsiveness
PkNF-YC 2	1304	6	light responsiveness
PkNF-YC 2	1841	8	light responsiveness
PkNF-YC 2	709	4	light responsiveness
PkNF-YC 2	709	4	light responsiveness
PkNF-YC 2	336	10	light responsiveness
PkNF-YC 2	1597	6	light responsiveness
PkNF-YC 2	1983	6	low-temperature responsiveness
PkNF-YC 2	232	6	MeJA responsiveness
PkNF-YC 2	302	6	MeJA responsiveness
PkNF-YC 2	626	6	MeJA responsiveness
PkNF-YC 2	680	8	MeJA responsiveness
PkNF-YC 2	1559	6	MeJA responsiveness
PkNF-YC 2	1352	6	MeJA responsiveness
PkNF-YC 2	145.5	15	meristem expression
PkNF-YC 3	54	4	abscisic acid responsiveness
PkNF-YC 3	46	12	abscisic acid responsiveness
PkNF-YC 3	52	6	abscisic acid responsiveness
PkNF-YC 3	1617	10	anaerobic induction element
PkNF-YC 3	1119	8	anaerobic induction element
PkNF-YC 3	1938	6	anaerobic induction element
PkNF-YC 3	52	6	defense and stress responsiveness
PkNF-YC 3	819	10	endosperm expression element
PkNF-YC 3	1186	6	light responsiveness
PkNF-YC 3	1002	6	light responsiveness
PkNF-YC 3	1008	6	light responsiveness
PkNF-YC 3	1292	6	light responsiveness
PkNF-YC 3	1601	6	light responsiveness
PkNF-YC 3	52	6	light responsiveness

PkNF-YC 3	245	6	light responsiveness
PkNF-YC 3	250	10	light responsiveness
PkNF-YC 3	1557	10	light responsiveness
PkNF-YC 3	326	8	light responsiveness
PkNF-YC 3	377	8	light responsiveness
PkNF-YC 3	861	6	light responsiveness
PkNF-YC 3	1436	14	light responsiveness
PkNF-YC 3	1776	14	light responsiveness
PkNF-YC 3	542	6	MeJA responsiveness
PkNF-YC 4	1967	14	anaerobic induction element
PkNF-YC 4	1269	6	anaerobic induction element
PkNF-YC 4	252	12	anaerobic induction element
PkNF-YC 4	781	6	anaerobic induction element
PkNF-YC 4	713	4	auxin responsiveness
PkNF-YC 4	381	4	auxin responsiveness
PkNF-YC 4	165	6	drought responsiveness
PkNF-YC 4	575	6	light responsiveness
PkNF-YC 4	1617	6	light responsiveness
PkNF-YC 4	1472	8	light responsiveness
PkNF-YC 4	381	4	light responsiveness
PkNF-YC 4	418	4	light responsiveness
PkNF-YC 4	418	4	light responsiveness
PkNF-YC 4	713	4	light responsiveness
PkNF-YC 4	1929	8	light responsiveness
PkNF-YC 4	304	6	light responsiveness
PkNF-YC 4	149	6	light responsiveness
PkNF-YC 4	102	8	light responsiveness
PkNF-YC 4	1352	6	light responsiveness
PkNF-YC 4	1973	10	low-temperature responsiveness
PkNF-YC 4	891	10	MeJA responsiveness
PkNF-YC 4	636	6	seed-specific regulation
PkNF-YC 5	867	14	anaerobic induction element
PkNF-YC 5	706	6	drought responsiveness

PkNF-YC 5	466	8	elements of regulating flavonoid biosynthesis
PkNF-YC 5	20	4	light responsiveness
PkNF-YC 5	76	12	light responsiveness
PkNF-YC 5	570	8	light responsiveness
PkNF-YC 5	281	6	low-temperature responsiveness
PkNF-YC 5	19	6	MeJA responsiveness
PkNF-YC 6	107	12	abscisic acid responsiveness
PkNF-YC 6	604	6	abscisic acid responsiveness
PkNF-YC 6	772	6	abscisic acid responsiveness
PkNF-YC 6	110	6	anaerobic induction element
PkNF-YC 6	386	10	anaerobic induction element
PkNF-YC 6	1681	6	anaerobic induction element
PkNF-YC 6	41	6	auxin responsiveness
PkNF-YC 6	186	6	auxin responsiveness
PkNF-YC 6	401	10	auxin responsiveness
PkNF-YC 6	1513	6	cycle regulation element
PkNF-YC 6	700	8	gibberellin responsiveness
PkNF-YC 6	1475	6	light responsiveness
PkNF-YC 6	1395	12	light responsiveness
PkNF-YC 6	1725	4	light responsiveness
PkNF-YC 6	1791	4	light responsiveness
PkNF-YC 6	1840	4	light responsiveness
PkNF-YC 6	1406	6	low-temperature responsiveness
PkNF-YC 6	12	6	low-temperature responsiveness
PkNF-YC 6	558	6	low-temperature responsiveness
PkNF-YC 6	1725	4	MeJA responsiveness
PkNF-YC 6	1791	4	MeJA responsiveness
PkNF-YC 6	1840	4	MeJA responsiveness
PkNF-YC 6	298	6	seed-specific regulation
PkNF-YC 7	186	6	abscisic acid responsiveness
PkNF-YC 7	1664	8	anaerobic induction element
PkNF-YC 7	647	6	anaerobic induction element
PkNF-YC 7	1895	8	anaerobic induction element

PkNF-YC 7	388	6	auxin responsiveness
PkNF-YC 7	-2	4	auxin responsiveness
PkNF-YC 7	316	6	circadian control element
PkNF-YC 7	880	6	defense and stress responsiveness
PkNF-YC 7	1723	6	defense and stress responsiveness
PkNF-YC 7	495	10	gibberellin responsiveness
PkNF-YC 7	1517	12	light responsiveness
PkNF-YC 7	661	12	light responsiveness
PkNF-YC 7	1253	12	light responsiveness
PkNF-YC 7	293	4	light responsiveness
PkNF-YC 7	656	4	light responsiveness
PkNF-YC 7	1182	10	light responsiveness
PkNF-YC 7	445	6	light responsiveness
PkNF-YC 7	343	6	light responsiveness
PkNF-YC 7	932	10	light responsiveness
PkNF-YC 7	1115	10	light responsiveness
PkNF-YC 7	507	12	light responsiveness
PkNF-YC 7	1369	6	low-temperature responsiveness
PkNF-YC 7	-3	6	MeJA responsiveness
PkNF-YC 7	293	4	MeJA responsiveness
PkNF-YC 7	656	4	MeJA responsiveness
PkNF-YC 7	1090	6	MeJA responsiveness
PkNF-YC 7	1520	6	MeJA responsiveness
PkNF-YC 7	1548	6	MeJA responsiveness
PkNF-YC 7	1405	6	salicylic acid responsiveness
PkNF-YC 7	1684	12	zein metabolism regulation

Table S8. The annotion of the interacting proteins.

ID	NR tophit name	Swiss_tophit_name	Swissprot tophit descrip
Pkor01G00025	ABK25822.1	sp|Q9SLA8|FABI_ARATH	Enoyl-[acyl-carrier-protein] reductase [NADH], chloroplastic
Pkor01G00360	ERN03715.1	sp|Q9XI07|SWI3C_ARATH	SWI/SNF complex subunit SWI3C
Pkor01G00537	PTQ32541.1	sp|F4KD38|NRPC2_ARATH	DNA-directed RNA polymerase III subunit 2
Pkor01G01140	ABK23141.1	sp|O64645|SOC1_ARATH	MADS-box protein SOC1
Pkor01G01596	ABK26041.1	sp|Q9M2B4|ALFL3_ARATH	PHD finger protein ALFIN-LIKE 3
Pkor01G01654	PTQ42796.1	sp|Q5BJY3|IN80C_RAT	INO80 complex subunit C
Pkor01G01888	ADE76806.1	sp|Q9C670|BH076_ARATH	Transcription factor bHLH76
Pkor01G01931	XP_020113267.1	sp|Q680U9|TFB2_ARATH	General transcription and DNA repair factor IIH subunit TFB2
Pkor01G02160	PNR43620.1	sp|Q8GYD9|SDE3_ARATH	Probable RNA helicase SDE3
Pkor01G02467	ERN02236.1	sp|Q940B8|KN13A_ARATH	Kinesin-like protein KIN-13A
Pkor01G02871	ACN40595.1	sp|Q2QY04|TAP46_ORYSJ	PP2A regulatory subunit TAP46
Pkor01G03054	XP_010268664.1	sp|F4JNY0|APE2_ARATH	DNA-(apurinic or apyrimidinic site) lyase 2
Pkor02G00834	PTQ41170.1	sp|Q9SII0|H2AV2_ARATH	Probable histone H2A variant 2
Pkor02G01024	AFV78821.1	sp|Q689G9|PRR1_ORYSJ	Two-component response regulator-like PRR1
Pkor02G01245	PNR35253.1	sp|Q8LC79|GAT18_ARATH	GATA transcription factor 18
Pkor02G01391	XP_028079717.1	sp|Q9LL84|DNLI4_ARATH	DNA ligase 4
Pkor02G01440	XP_024390852.1	sp|Q9WU42|NCOR2_MOUSE	Nuclear receptor corepressor 2
Pkor02G01970	PNR60189.1	sp|Q9ZWA6|IDD3_ARATH	Zinc finger protein MAGPIE
Pkor02G02173	ANC94891.1	sp|Q6X7J5|WOX8_ARATH	WUSCHEL-related homeobox 8
Pkor02G02527	PTQ39354.1	_	_
Pkor03G00090	ABR17676.1	sp|Q9AR19|GCN5_ARATH	Histone acetyltransferase GCN5
Pkor03G00197	XP_015889734.1	sp|O48832|ERD7_ARATH	Protein EARLY-RESPONSIVE TO DEHYDRATION 7, chloroplastic
Pkor03G00252	ABK25980.1	sp|Q9FFF5|ALFL1_ARATH	PHD finger protein ALFIN-LIKE 1
Pkor03G00920	XP_024517508.1	sp|Q54R14|Y3443_DICDI	SET domain-containing protein DDB_G0283443
Pkor03G01113	ACN40350.1	sp|Q42808|TBP_SOYBN	TATA-box-binding protein OS=Glycine max
Pkor03G01331	PTQ27808.1	sp|Q9SBJ1|PDK_ARATH	[Pyruvate dehydrogenase (acetyl-transferring)] kinase, mitochondrial
Pkor03G01938	XP_010943344.1	sp|Q4UM42|TOP1_RICFE	DNA topoisomerase 1 OS=Rickettsia felis (strain ATCC VR-1525 / URRWXCal2)
Pkor03G02296	OVA04383.1	sp|Q8LFS6|BRN1L_ARATH	RNA-binding protein BRN1 OS=Arabidopsis thaliana
Pkor03G02350	XP_004491513.1	_	_
Pkor03G02600	PTQ49926.1	sp|Q54IR8|WASC5_DICDI	WASH complex subunit 5
Pkor03G02667	PTQ49466.1	sp|Q8L3Z8|FZR2_ARATH	Protein FIZZY-RELATED 2

Pkor04G00506	PNR62333.1	sp|Q9ZWA6|IDD3_ARATH	Zinc finger protein MAGPIE
Pkor04G00773	ABK24137.1	sp|Q84JP1|NFYA7_ARATH	Nuclear transcription factor Y subunit A-7
Pkor04G01141	ABK26131.1	_	_
Pkor04G01275	PNR29741.1	sp|Q941B6|UBC37_ARATH	Probable ubiquitin-conjugating enzyme E2
Pkor04G01300	ABR16284.1	_	_
Pkor04G01469	ERN19683.1	sp|P53492|ACT7_ARATH	Actin-7
Pkor04G01697	XP_019705765.1	sp|Q9Z0H0|CDC7_MOUSE	Cell division cycle 7-related protein kinase
Pkor04G01793	PTQ27137.1	sp|Q32SG5|RIK_MAIZE	Protein RIK
Pkor04G01856	ADE77365.1	sp|Q54IS6|RTF2_DICDI	Replication termination factor 2
Pkor04G01933	ACP19075.1	sp|P53492|ACT7_ARATH	Actin-7
Pkor04G02192	ABR16282.1	sp|Q9SFV2|FHA2_ARATH	FHA domain-containing protein FHA2
Pkor04G02204	XP_020525461.1	sp|Q9SL02|RAD50_ARATH	DNA repair protein RAD50
Pkor04G02543	PNR59617.1	sp|Q7F830|CCA11_ORYSJ	Cyclin-A1-1
Pkor05G00270	PNR51730.1	_	_
Pkor05G01413	ACN40356.1	sp|P35683|IF4A1_ORYSJ	Eukaryotic initiation factor 4A-1
Pkor05G01577	ATG70990.1	sp|Q9FF61|SM3L1_ARATH	Putative SWI/SNF-related matrix-associated actin-dependent regulator of chromatin subfamily A member 3-like 1
Pkor05G01591	ABK24544.1	sp|Q10NY2|TPR3_ORYSJ	Protein TPR3
Pkor05G01601	XP_010917272.1	sp|Q500V9|DPB2_ARATH	DNA polymerase epsilon subunit B
Pkor05G01717	ABR16132.1	sp|Q38861|XPB1_ARATH	General transcription and DNA repair factor IIH helicase subunit XPB1
Pkor05G01792	XP_031500233.1	sp|Q945S8|ASHH3_ARATH	Histone-lysine N-methyltransferase ASHH3
Pkor05G02429	ERN05631.1	sp|Q9LS09|ASF1B_ARATH	Histone chaperone ASF1B
Pkor06G00225	ERM99329.1	sp|Q6L4L4|SIZ1_ORYSJ	E3 SUMO-protein ligase SIZ1
Pkor06G00476	OVA17107.1	sp|Q42381|HLS1_ARATH	Probable N-acetyltransferase HLS1
Pkor06G00862	CAC27333.1	sp|Q147G5|CCA22_ARATH	Cyclin-A2-2
Pkor06G01303	OVA07048.1	sp|Q6Z2G9|TLP5_ORYSJ	Tubby-like F-box protein 5
Pkor06G01334	PTQ35570.1	sp|O88738|BIRC6_MOUSE	Baculoviral IAP repeat-containing protein 6
Pkor06G01586	XP_031482496.1	sp|Q9S775|PKL_ARATH	CHD3-type chromatin-remodeling factor PICKLE
Pkor06G01598	PNR55649.1	sp|F4JL28|EBS_ARATH	Chromatin remodeling protein EBS
Pkor06G01919	ACN40443.1	sp|Q940X7|RBX1A_ARATH	RING-box protein 1a
Pkor06G01964	ABK23801.1	_	_
Pkor06G02461	ABK26989.1	sp|Q5XEM9|ALFL5_ARATH	PHD finger protein ALFIN-LIKE 5
Pkor06G02478	ABK26838.1	sp|Q39027|MPK7_ARATH	Mitogen-activated protein kinase 7
Pkor07G00057	RWR88769.1	sp|F4I443|BARD1_ARATH	BRCA1-associated RING domain protein 1

Pkor07G00791	PTQ49443.1	sp|Q9LJG8|ASIL2_ARATH	Trihelix transcription factor ASIL2
Pkor07G01130	ERN17623.1	sp|O64827|SUVR5_ARATH	Histone-lysine N-methyltransferase SUVR5
Pkor07G01229	ABR18264.1	sp|Q9ZT29|SC5D_TOBAC	Delta(7)-sterol-C5(6)-desaturase
Pkor07G01329	XP_024370825.1	_	_
Pkor07G01457	XP_017697874.1	sp|Q5W9E7|MUS81_ARATH	Crossover junction endonuclease MUS81
Pkor07G01522	ABR17799.1	sp|Q8RXY6|SG29A_ARATH	SAGA-associated factor 29 homolog A
Pkor07G01724	ADE75910.1	sp|O65499|ZAT3_ARATH	Zinc finger protein ZAT3
Pkor07G02417	PNR48581.1	sp|Q9SVY1|ZWIP2_ARATH	Zinc finger protein WIP2
Pkor07G02418	PNR48581.1	sp|Q9FX68|ZWIP6_ARATH	Zinc finger protein WIP6
Pkor08G00452	PTQ50472.1	sp|Q9LKR8|RAF1_ARATH	Rubisco accumulation factor 1.1, chloroplastic
Pkor08G00651	QHG11442.1	sp|Q9C7U7|MYB20_ARATH	Transcription factor MYB20
Pkor08G00717	ABK24385.1	sp|P49572|TRPC_ARATH	Indole-3-glycerol phosphate synthase, chloroplastic
Pkor08G00986	PNR38197.1	sp|Q9FMR9|RIN1_ARATH	RuvB-like protein 1
Pkor08G01491	ADE77185.1	sp|Q9LMK5|STN1_ARATH	CST complex subunit STN1
Pkor08G01800	PTQ45728.1	sp|Q0J0S6|TOP3B_ORYSJ	DNA topoisomerase 3-beta
Pkor08G01821	XP_019073139.1	sp|Q94BR5|CHR28_ARATH	Helicase-like transcription factor CHR28
Pkor08G02402	ERN14111.1	sp|Q9ZPY9|P4KG4_ARATH	Phosphatidylinositol 4-kinase gamma 4
Pkor08G02430	ERN20534.1	sp|Q9C6Y3|CCA11_ARATH	Cyclin-A1-1
Pkor09G00141	ERN06336.1	sp|Q8H252|RBR_COCNU	Retinoblastoma-related protein
Pkor09G00261	PNR51577.1	sp|Q9MAT6|HMG15_ARATH	High mobility group B protein 15
Pkor09G00465	AGV07536.1	sp|Q9FM03|DOF56_ARATH	Dof zinc finger protein DOF5.6
Pkor09G01522	ERN13745.1	sp|Q10RP4|SE14_ORYSJ	Lysine-specific demethylase SE14
Pkor09G01716	XP_019708666.1	sp|Q9XGM2|MRE11_ARATH	Double-strand break repair protein MRE11
Pkor09G01740	ABK26256.1	sp|K7K424|DAT2D_SOYBN	Diacylglycerol O-acyltransferase 2D
Pkor09G01769	ERN11522.1	sp|Q940Y3|ARID3_ARATH	AT-rich interactive domain-containing protein 3
Pkor09G01923	ADE76155.1	sp|Q6K8X6|ORR23_ORYSJ	Two-component response regulator ORR23
Pkor09G02055	ABK24942.1	sp|Q9FIC3|BOLA2_ARATH	Protein BOLA2
Pkor09G02179	ADE77570.1	sp|Q39211|NRPB3_ARATH	DNA-directed RNA polymerases II, IV and V subunit 3
Pkor09G02218	AHX56184.1	sp|Q84JP1|NFYA7_ARATH	Nuclear transcription factor Y subunit A-7
Pkor09G02226	PNR55481.1	sp|Q943I6|STOP1_ORYSJ	Zinc finger protein STOP1 homolog
Pkor10G00043	AFG56533.1	sp|F0NBH8|KMT_SULIR	Protein-lysine N-methyltransferase
Pkor10G00639	ERN02249.1	sp|Q6ZQK0|CNDD3_MOUSE	Condensin-2 complex subunit D3
Pkor10G00743	ABR18461.1	sp|Q42712|FATA_CORSA	Oleoyl-acyl carrier protein thioesterase, chloroplastic (Fragment)

Pkor10G00869	AAG22585.1	sp|P26307|VIV1_MAIZE	Regulatory protein viviparous-1
Pkor10G00969	CBI33371.3	sp|Q84WJ2|PRI2_ARATH	Probable DNA primase large subunit
Pkor10G01405	CAC84681.1	sp|P35063|H2AX_PICAB	Histone H2AX
Pkor10G01578	XP_020082741.1	sp|Q0J7U6|TPR2_ORYSJ	Protein TOPLESS-RELATED PROTEIN 2
Pkor10G01780	XP_011628169.1	sp|Q93Z16|RPN2_ARATH	Dolichyl-diphosphooligosaccharide--protein glycosyltransferase subunit 2
Pkor10G01984	ACN40963.1	sp|A9TF79|SRK2A_PHYPA	Serine/threonine-protein kinase SRK2A
Pkor10G02012	AHW42470.1	sp|Q8S4W7|GAI1_VITVI	DELLA protein GAI1
Pkor11G00402	XP_020528358.1	sp|A8MS85|SPT61_ARATH	Transcription elongation factor SPT6 homolog
Pkor11G00426	XP_031482098.1	sp|Q6DRL5|MBB1A_DANRE	Myb-binding protein 1A-like protein
Pkor11G00593	PNR58335.1	sp|O81221|ACT_GOSHI	Actin OS=Gossypium hirsutum
Pkor11G00637	ABK26855.1	sp|Q14191|WRN_HUMAN	Werner syndrome ATP-dependent helicase
Pkor11G00698	ERN01997.1	sp|Q9SBJ1|PDK_ARATH	[Pyruvate dehydrogenase (acetyl-transferring)] kinase, mitochondrial
Pkor11G01151	ABR17842.1	sp|Q94AH6|CUL1_ARATH	Cullin-1
Pkor11G01161	PNR58184.1	_	_
Pkor11G01373	PNR35265.1	sp|Q9FPT5|UBP1_ARATH	Ubiquitin carboxyl-terminal hydrolase 1
Pkor11G01417	XP_008805740.1	sp|Q946J8|LHP1_ARATH	Chromo domain-containing protein LHP1
Pkor11G01427	OVA03206.1	sp|F4KBP5|CHR4_ARATH	Protein CHROMATIN REMODELING 4
Pkor11G01536	ABR17188.1	sp|Q8R050|ERF3A_MOUSE	Eukaryotic peptide chain release factor GTP-binding subunit ERF3A
Pkor11G01887	PTQ32107.1	sp|A0A1P8AS03|ECT4_ARATH	YTH domain-containing protein ECT4
Pkor11G01895	AIZ74347.1	sp|P26356|TBP1_WHEAT	TATA-box-binding protein 1
Pkor11G01896	ABR18447.1	sp|Q03684|BIP4_TOBAC	Luminal-binding protein 4
Pkor11G02534	PNR37979.1	_	_
Pkor12G00567	PTQ34413.1	sp|Q6NRE8|SUV91_XENLA	Histone-lysine N-methyltransferase SUV39H1
Pkor12G00613	ADE76569.1	sp|Q9Y2Y1|RPC10_HUMAN	DNA-directed RNA polymerase III subunit RPC10
Pkor12G00648	ERN13085.1	sp|Q652L2|HIRA_ORYSJ	Protein HIRA
Pkor12G01081	ABK25222.1	sp|Q9M077|AUR1_ARATH	Serine/threonine-protein kinase Aurora-1
Pkor12G01223	AIZ74323.1	sp|O81221|ACT_GOSHI	Actin OS=Gossypium hirsutum
Pkor12G01240	AJA90781.1	sp|Q9LQ02|NRPD1_ARATH	DNA-directed RNA polymerase IV subunit 1
Pkor12G01514	ERN02605.1	sp|Q6NLW5|XRI1_ARATH	Protein XRI1
Pkor12G01722	AOZ15520.1	sp|Q9SQ57|PXG_SESIN	Peroxygenase
Pkor12G01969	ABR18038.1	_	_
Pkor12G01972	PTQ33700.1	sp|P51566|AFC1_ARATH	Serine/threonine-protein kinase AFC1
Pkor12G02112	PNR62471.1	sp|Q9U7D1|H3_MASBA	Histone H3

Table S9. The node ID of the interacting proteins.

Node1	Node2
NF-YB 2	Pkor01G00537
NF-YB 2	Pkor02G00716
NF-YB 2	Pkor02G01440
NF-YB 2	Pkor02G01970
NF-YB 2	Pkor02G02527
NF-YB 2	Pkor03G02667
NF-YB 2	Pkor04G02096
NF-YB 2	Pkor05G00270
NF-YB 2	Pkor06G01598
NF-YB 2	Pkor06G02478
NF-YB 2	Pkor07G01724
NF-YB 2	Pkor07G02418
NF-YB 2	Pkor08G00452
NF-YB 2	Pkor09G00261
NF-YB 2	Pkor09G01347
NF-YB 2	Pkor09G02055
NF-YB 2	Pkor09G02226
NF-YB 2	Pkor10G01405
NF-YB 2	Pkor11G00593
NF-YB 2	Pkor11G01417
NF-YB 2	Pkor11G01887
NF-YB 2	Pkor11G01895
NF-YB 2	Pkor11G02534
NF-YB 2	Pkor12G00032
NF-YB 2	Pkor12G00567
NF-YB 2	Pkor12G01972
NF-YB 3	NF-YC 3
NF-YB 3	Pkor01G00537
NF-YB 3	Pkor01G01654
NF-YB 3	Pkor02G00716

NF-YB 3	Pkor02G00811
NF-YB 3	Pkor02G00834
NF-YB 3	Pkor02G01245
NF-YB 3	Pkor02G01440
NF-YB 3	Pkor02G01970
NF-YB 3	Pkor02G02527
NF-YB 3	Pkor03G01331
NF-YB 3	Pkor03G01576
NF-YB 3	Pkor03G02667
NF-YB 3	Pkor04G00506
NF-YB 3	Pkor04G00773
NF-YB 3	Pkor04G01141
NF-YB 3	Pkor04G01275
NF-YB 3	Pkor04G01933
NF-YB 3	Pkor04G02096
NF-YB 3	Pkor05G00270
NF-YB 3	Pkor05G01413
NF-YB 3	Pkor06G00862
NF-YB 3	Pkor06G01334
NF-YB 3	Pkor06G01598
NF-YB 3	Pkor06G02478
NF-YB 3	Pkor07G01724
NF-YB 3	Pkor07G02417
NF-YB 3	Pkor07G02418
NF-YB 3	Pkor08G00452
NF-YB 3	Pkor08G00986
NF-YB 3	Pkor08G01800
NF-YB 3	Pkor09G00261
NF-YB 3	Pkor09G01347
NF-YB 3	Pkor09G01923
NF-YB 3	Pkor09G02055
NF-YB 3	Pkor09G02226
NF-YB 3	Pkor10G00043

NF-YB 3	Pkor10G01405
NF-YB 3	Pkor11G00593
NF-YB 3	Pkor11G01417
NF-YB 3	Pkor11G01536
NF-YB 3	Pkor11G01887
NF-YB 3	Pkor11G01895
NF-YB 3	Pkor11G02534
NF-YB 3	Pkor12G00032
NF-YB 3	Pkor12G00567
NF-YB 3	Pkor12G01969
NF-YB 3	Pkor12G01971
NF-YB 3	Pkor12G01972
NF-YB 5	Pkor01G00360
NF-YB 5	Pkor01G01140
NF-YB 5	Pkor01G01596
NF-YB 5	Pkor01G02467
NF-YB 5	Pkor05G01792
NF-YB 5	Pkor06G00225
NF-YB 5	Pkor07G01229
NF-YB 5	Pkor09G02218
NF-YB 5	Pkor10G00639
NF-YB 5	Pkor11G01607
NF-YB 5	Pkor11G01896
NF-YB 5	Pkor12G00085
NF-YB 5	Pkor12G01514
NF-YB 7	Pkor01G00025
NF-YB 7	Pkor01G00360
NF-YB 7	Pkor01G01596
NF-YB 7	Pkor01G02467
NF-YB 7	Pkor02G00810
NF-YB 7	Pkor03G00252
NF-YB 7	Pkor03G02350
NF-YB 7	Pkor04G01300

NF-YB 7	Pkor04G02192
NF-YB 7	Pkor04G02204
NF-YB 7	Pkor05G01792
NF-YB 7	Pkor05G02429
NF-YB 7	Pkor06G00225
NF-YB 7	Pkor06G01586
NF-YB 7	Pkor06G02461
NF-YB 7	Pkor07G00791
NF-YB 7	Pkor07G01130
NF-YB 7	Pkor07G01229
NF-YB 7	Pkor07G01522
NF-YB 7	Pkor08G00886
NF-YB 7	Pkor08G02402
NF-YB 7	Pkor09G00141
NF-YB 7	Pkor09G01522
NF-YB 7	Pkor09G01740
NF-YB 7	Pkor09G01769
NF-YB 7	Pkor09G02179
NF-YB 7	Pkor09G02218
NF-YB 7	Pkor10G00639
NF-YB 7	Pkor10G00743
NF-YB 7	Pkor10G00869
NF-YB 7	Pkor11G00426
NF-YB 7	Pkor11G00698
NF-YB 7	Pkor11G01896
NF-YB 7	Pkor12G00085
NF-YB 7	Pkor12G00648
NF-YB 7	Pkor12G01081
NF-YB 7	Pkor12G01722
NF-YC 2	Pkor03G02296
NF-YC 2	Pkor11G01427
NF-YC 3	NF-YB 2
NF-YC 3	Pkor02G02397

NF-YC 3	Pkor02G02574
NF-YC 3	Pkor06G01964
NF-YC 3	Pkor07G01329
NF-YC 3	Pkor09G02055
NF-YC 3	Pkor10G01984
NF-YC 3	Pkor11G00637
NF-YC 3	Pkor11G01887
NF-YC 3	Pkor11G01895
NF-YC 4	Pkor02G01024
NF-YC 4	Pkor10G02012
NF-YC 5	Pkor01G03054
NF-YC 5	Pkor02G01391
NF-YC 5	Pkor08G01821
NF-YC 5	Pkor09G00465
NF-YC 5	Pkor10G00969
NF-YC 7	Pkor03G00090
NF-YC 7	Pkor03G01938
NF-YC 7	Pkor04G01697
NF-YC 7	Pkor05G01577
NF-YC 7	Pkor05G01601
NF-YC 7	Pkor05G02007
NF-YC 7	Pkor06G01919
NF-YC 7	Pkor07G01457
NF-YC 7	Pkor08G01491
NF-YC 7	Pkor10G01578
Pkor01G01654	NF-YB 2
Pkor01G01888	NF-YC 7
Pkor01G01931	NF-YC 2
Pkor01G02160	NF-YB 2
Pkor01G02160	NF-YB 3
Pkor01G02160	NF-YC 3
Pkor01G02871	NF-YC 7
Pkor02G00810	NF-YB 5

Pkor02G00811	NF-YB 2
Pkor02G00811	NF-YC 3
Pkor02G00834	NF-YB 2
Pkor02G01245	NF-YB 2
Pkor02G02173	NF-YB 6
Pkor03G00197	NF-YB 5
Pkor03G00252	NF-YB 5
Pkor03G00920	NF-YB 2
Pkor03G00920	NF-YB 3
Pkor03G01113	NF-YB 5
Pkor03G01113	NF-YB 7
Pkor03G01331	NF-YB 2
Pkor03G01576	NF-YB 2
Pkor03G01576	NF-YC 3
Pkor03G02350	NF-YB 5
Pkor03G02600	NF-YC 3
Pkor04G00506	NF-YB 2
Pkor04G00773	NF-YB 2
Pkor04G00773	NF-YC 3
Pkor04G01141	NF-YB 2
Pkor04G01275	NF-YB 2
Pkor04G01275	NF-YC 3
Pkor04G01300	NF-YB 5
Pkor04G01469	NF-YB 5
Pkor04G01793	NF-YB 5
Pkor04G01856	NF-YB 5
Pkor04G01933	NF-YB 2
Pkor04G02192	NF-YB 5
Pkor04G02204	NF-YB 5
Pkor04G02543	NF-YB 2
Pkor04G02543	NF-YB 3
Pkor05G01413	NF-YB 2
Pkor05G01591	NF-YB 2

Pkor05G01591	NF-YB 3
Pkor05G01591	NF-YC 3
Pkor05G01717	NF-YC 2
Pkor05G02429	NF-YB 5
Pkor06G00476	NF-YC 2
Pkor06G00862	NF-YB 2
Pkor06G01303	NF-YC 2
Pkor06G01334	NF-YB 2
Pkor06G01334	NF-YC 3
Pkor06G02461	NF-YB 5
Pkor07G00057	NF-YC 7
Pkor07G01130	NF-YB 5
Pkor07G01522	NF-YB 5
Pkor07G02417	NF-YB 2
Pkor08G00271	NF-YC 3
Pkor08G00651	NF-YC 7
Pkor08G00717	NF-YC 3
Pkor08G00886	NF-YB 5
Pkor08G00986	NF-YB 2
Pkor08G01800	NF-YB 2
Pkor08G01800	NF-YC 3
Pkor08G02402	NF-YB 5
Pkor08G02430	NF-YB 5
Pkor09G00141	NF-YB 5
Pkor09G01522	NF-YB 5
Pkor09G01716	NF-YC 7
Pkor09G01769	NF-YB 5
Pkor09G01923	NF-YB 2
Pkor09G02179	NF-YB 5
Pkor10G00043	NF-YB 2
Pkor10G00504	NF-YC 7
Pkor10G00869	NF-YB 5
Pkor10G01780	NF-YB 5

Pkor11G00402	NF-YB 5
Pkor11G00426	NF-YB 5
Pkor11G01151	NF-YC 7
Pkor11G01161	NF-YC 3
Pkor11G01373	NF-YC 3
Pkor11G01536	NF-YB 2
Pkor12G00613	NF-YB 2
Pkor12G00613	NF-YB 3
Pkor12G00648	NF-YB 5
Pkor12G01081	NF-YB 5
Pkor12G01183	NF-YC 2
Pkor12G01223	NF-YB 5
Pkor12G01240	NF-YB 5
Pkor12G01240	NF-YB 7
Pkor12G01969	NF-YB 2
Pkor12G01971	NF-YB 2
Pkor12G02112	NF-YC 3

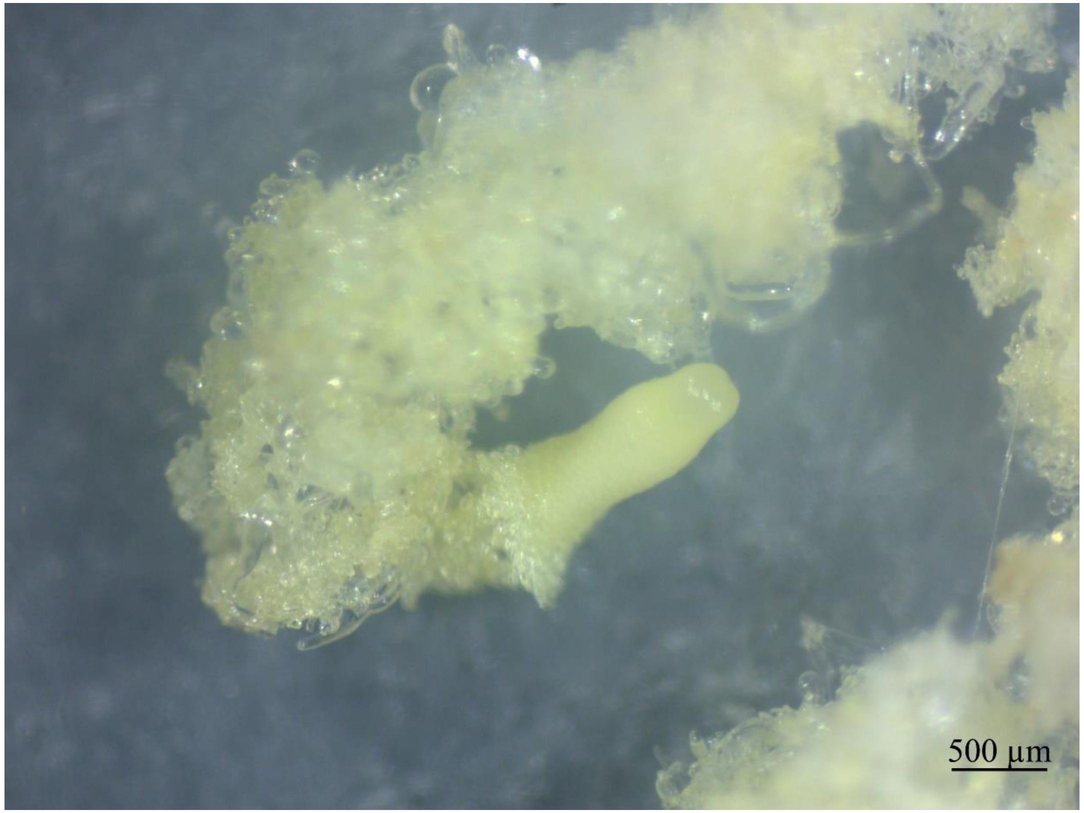

Figure S1. Early cotyledon type embryo from transgenic somatic embryo.

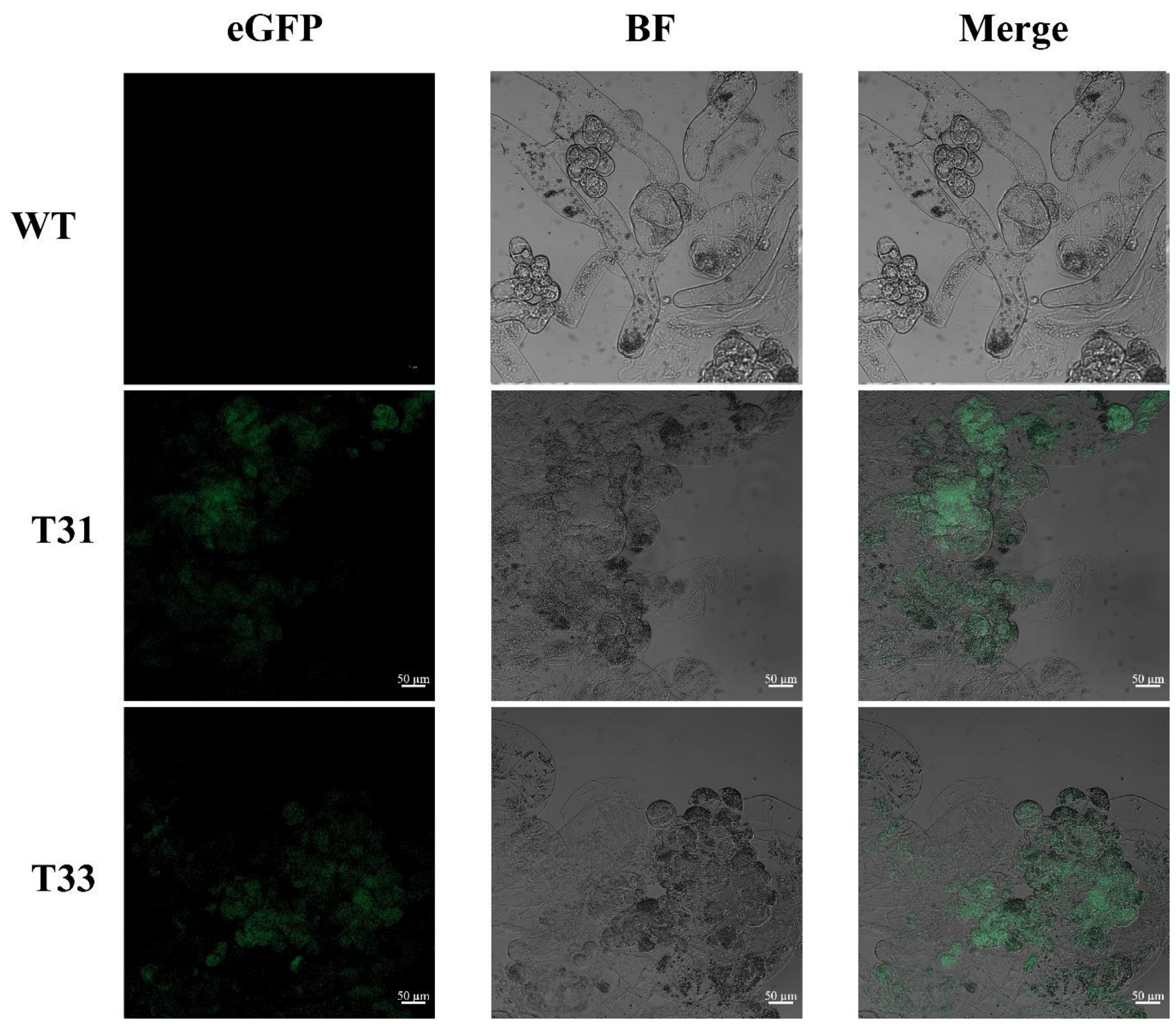

Figure S2. GFP fluorescence observation of wild-type and transgenic embryonic callus tissues.
